# Evaluating the Effectiveness of Apps Designed to Reduce Mobile Phone Use and Prevent Maladaptive Mobile Phone Use: Multimethod Study

**DOI:** 10.2196/42541

**Published:** 2023-08-29

**Authors:** Fety Ilma Rahmillah, Amina Tariq, Mark King, Oscar Oviedo-Trespalacios

**Affiliations:** 1 Faculty of Health Queensland University of Technology Brisbane Australia; 2 Faculty of Technology, Policy and Management Delft University of Technology Delft Netherlands

**Keywords:** mobile phone, maladaptive mobile phone use, apps, features, problematic phone use

## Abstract

**Background:**

Mobile apps are a popular strategy for reducing mobile phone use and preventing maladaptive mobile phone use (MMPU). Previous research efforts have been made to understand the features of apps that have the potential to reduce mobile phone use and MMPU. However, there has been a lack of a comprehensive examination of the effectiveness of such apps and their features.

**Objective:**

This paper investigated existing apps designed to reduce mobile phone use and prevent MMPU and examined the evidence of their effectiveness. The research aimed to provide a comprehensive analysis of app features that can reduce mobile phone use and MMPU, while also assessing their effectiveness. In addition, we explored users’ perceptions of these apps and the various features the apps offer to understand potential adoption issues and identify opportunities.

**Methods:**

This study used 3 methods: a review of scientific evidence, content analysis, and sentiment analysis.

**Results:**

Our study comprehensively examine the common features of 13 apps designed to reduce mobile phone use. We extracted and classified the features into 7 types: self-tracking, social tracking, goal setting, blocking, gamification, simplification, and assessment. The effectiveness of these apps in reducing mobile phone use and MMPU varied from weak to strong. On the basis of content analysis, self-tracking and goal setting were the most frequently used features, whereas gamification and assessment were used the least frequently. The intervention strategies that effectively reduce mobile phone use and MMPU included using grayscale mode, app limit features, and mixed interventions. Overall, users tended to accept these apps, as indicated by sentiment scores ranging from 61 to 86 out of 100.

**Conclusions:**

This study demonstrates that app-based management has the potential to reduce mobile phone use and MMPU. However, further research is required to evaluate the effectiveness of app-based interventions. Collaborations among researchers, app developers, mobile phone manufacturers, and policy makers could enhance the process of delivering, evaluating, and optimizing apps aimed at reducing mobile phone use and MMPU.

## Introduction

### Background

Mobile phones are beneficial in many aspects of our lives, such as working and staying connected with our family and friends regardless of distance as well as helping to organize day-to-day activities. People generally have favorable perceptions regarding their mobile phones and their impact. However, an individual’s level of mobile phone use can also lead to adverse outcomes. Excessive mobile phone use is often referred to as maladaptive mobile phone use (MMPU), which occurs when use negatively interferes with individuals’ work and social interactions [1,2]. MMPU encompasses various related concepts, including problematic mobile phone use, smartphone addiction, fear of missing out (FoMO; a persistent desire to share others’ gratifying experiences), nomophobia (the fear of being without a mobile phone), mobile phone involvement, compulsive mobile phone checking, texting dependency, texting automaticity (texting without thinking or intending to do it), and mobile phone dependency [2]. An example of how MMPU can result in adverse outcomes is the association between MMPU and risky mobile phone use behaviors, such as falling, slipping, bumps or collisions, moving violations, road traffic injuries, and motor vehicle crashes [2,3]. The higher the MMPU score, the more likely road users are to engage with their mobile phones in risky situations, such as while driving, riding a motorcycle, cycling, or crossing the road [2]. Other issues linked to MMPU include decreased academic performance (measured in terms of grade point average) [4,5], increased anxiety [4], increased stress [4-6], and irregular sleep patterns [6]. The negative consequences of MMPU and its growing prevalence worldwide [1] highlight the need to identify effective strategies to manage and reduce MMPU.

One strategy to manage and reduce MMPU is to use apps designed specifically for reducing and regulating mobile phone use [7]; for instance, apps such as *AppDetox* can regulate information flow by setting rules, *RescueTime* can establish goals, and *Forest* uses gamification [8]. These apps can assist in reducing mobile phone use and MMPU. Evidence suggests that such apps can effectively limit overall mobile phone use and decrease perceived distractions [9]. Empirical studies have demonstrated that avoiding persuasive functions (resulting in a reduction of up to 37%) or disabling certain persuasive functions (resulting in a reduction of up to 16.72%) can effectively decrease mobile phone use [10]. As for built-in functions, enabling grayscale mode can reduce the desire to use the mobile phone because the interface becomes visually less appealing [11]. The blue light emitted from the colorful screen of the mobile phone can attract the human brain and trigger the release of cortisol, a hormone that promotes wakefulness [12]. Switching the mobile phone interface to grayscale mode eliminates positive reinforcements and can diminish the urge to stay engaged in social media [12]. However, apps that aim to manage and reduce mobile phone use and MMPU have faced criticism for not effectively changing habits and not being restrictive enough. As a result, doubts persist regarding the effectiveness of such apps in altering mobile phone use behavior [13]. A critical gap in the existing literature concerns understanding the effectiveness of such apps and their features in managing and reducing mobile phone use and MMPU.

There have been some previous attempts to understand the features of apps that have the potential to reduce mobile phone use and MMPU [8], such as through blocking [14,15], mobile phone vibration [16], tracking [14,17], goal advancement [14,17], group-based intervention [9,18], and gamification [19]. Such categorization of app features is essential because each feature could support different cognitive processes [20]. A classification of apps and browser extensions for digital self-control resulted in 4 features based on functionality: block or removal, self-tracking, goal advancement, and reward or punishment [21]. A recent review of Google’s *Digital Wellbeing* apps delineated 2 main categories of features: self-monitoring and interventions [13].

### Objectives

This paper examined current apps designed to reduce mobile phone use and prevent MMPU. It investigated the features of these apps and evaluated the extent to which the benefits of these features are supported by evidence of their effectiveness in reducing MMPU. The approach taken involved developing a comprehensive map of the app features that have been assessed in the scientific literature, along with the corresponding evidence for their effectiveness. In addition, this study explored users’ perceptions of the apps and the various features these apps offer, aiming to understand potential acceptance issues. The following research questions (RQs) were addressed:

RQ1: Which apps have been scientifically evaluated?

RQ2: What features are offered by current apps to reduce mobile phone use and MMPU?

RQ3: What is the efficacy of these apps?

RQ4: How have users responded to these apps and their features in terms of popularity and reviews?

## Methods

### Overview

**Figure 1:**
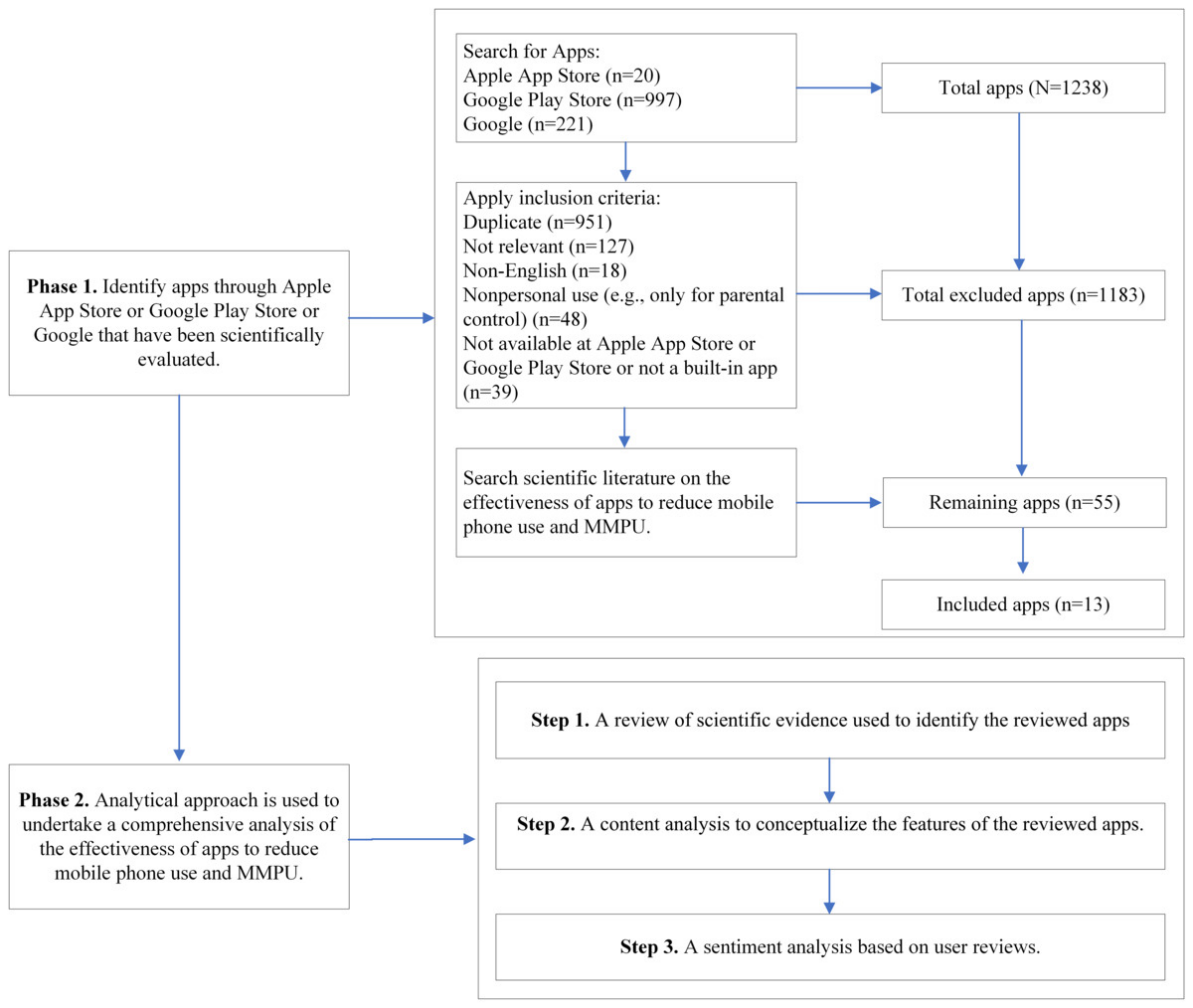
Methodology of the study. MMPU: maladaptive mobile phone use.

A multimethod approach was used to thoroughly address the aforementioned RQs, as depicted in Figure 1. The study encompassed 2 distinct phases. In the first phase, extensive web-based searches were conducted to identify a representative set of apps. The objective was to ensure a comprehensive selection of apps for further analysis. Subsequently, the second phase involved an analytical approach, encompassing a comprehensive examination of the effectiveness of the apps and their respective features in reducing mobile phone use and MMPU. This examination was achieved through a combination of literature review, content analysis, and sentiment analysis. Each of these steps will be elaborated upon in the subsequent sections of this paper, providing a detailed account of the methodology used in this study.

### Ethical Considerations

Before commencing the research, ethics approval for using secondary data was sought, and the study was deemed exempt by the human research ethics committee at Queensland University of Technology.

### Procedure to Identify Apps That Reduce Mobile Phone Use and MMPU

A web-based search was conducted to identify a representative set of apps that reduce mobile phone use and MMPU to be reviewed through the Google Play Store, the Apple App Store, and a Google search. A search through the first page of the Google search engine was needed to identify popular apps to be reviewed and to obtain comprehensive data regarding their aims and features as what has been done by [22]. The term “apps” was used as a generic reference encompassing functionalities offered by both built-in apps (eg, *Screen Time* [iOS] within Settings app, *Digital Wellbeing* [within Android]) and third-party apps downloaded from Apple App Store or Google Play Store, encompassing all software programs designed for reducing mobile phone use and MMPU on mobile phones. We used the following keywords: ((“[name of the potential app]” AND “app”) AND (“reduce” OR “limit”) AND (“screen time” OR “phone use”)). The original total number of apps was 1238. Next, the following exclusion criteria were applied: duplicate, not relevant (designed for computer or website, not mobile phone), non-English, nonpersonal use (eg, only for parental control), not available in the Google Play Store or Apple App Store, or not a built-in app. Of the 1238 apps, 55 (4.44%) were selected for further consideration after the application of the screening process based on the exclusion criteria. The next step involved identifying apps that have been evaluated in the scientific literature in terms of their effectiveness in supporting the reduction of mobile phone use and MMPU. To achieve this, each of the apps (n=55) was searched on Google Scholar, PubMed, and Scopus. Evidence of the efficacy of 13 (24%) of the 55 apps in 19 papers was identified through this process. Basic information about these apps is available in Multimedia Appendix 1.

### Analytical Approach

This section summarizes the analytical approach, including the research methods adopted to review scientific evidence on the effectiveness of the reviewed apps in reducing mobile phone use and MMPU, content analysis to extract app features, and sentiment analysis to examine users’ perceptions.

#### A Review of the Evidence Concerning the Effectiveness of Apps to Reduce Mobile Phone Use and MMPU

**Figure 2:**
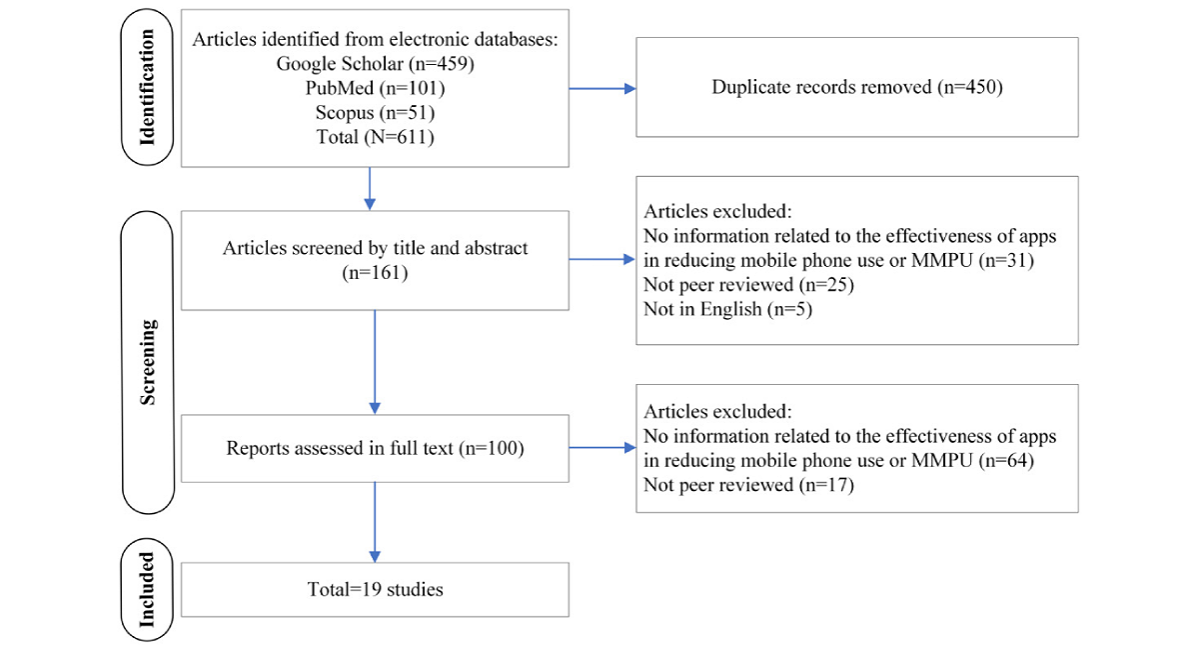
The PRISMA (Preferred Reporting Items for Systematic Reviews and Meta-Analyses) flowchart. MMPU: maladaptive mobile phone use.

This paper reviewed peer-reviewed literature concerning the evidence of apps to reduce mobile phone use and MMPU. The research papers concerning the original identified apps (n=55) were identified using Scopus, PubMed, and Google Scholar. As explained earlier, there was evidence about 13 (24%) of the 55 apps in 19 papers (refer to the *Results* section). Refer to Multimedia Appendix 2 for the details of the search strategy used in these databases. Time restrictions were not implemented, but only papers published before April 10, 2023, were considered. The inclusion criteria were as follows: (1) papers that provide information related to the effectiveness of apps and features in reducing mobile phone use and MMPU; (2) peer-reviewed scientific articles, including conference papers; and (3) papers published in English. The study was designed using preestablished criteria based on the PRISMA (Preferred Reporting Items for Systematic Reviews and Meta-Analyses) protocol (See Multimedia Appendix 3 for details) [23]. Papers were selected from the 3 aforementioned databases (Figure 2). A total of 611 papers were identified. Microsoft Excel was used for data extraction, and duplicates were removed manually by a team member (FIR). One team member (FIR) conducted the screening, which was verified by another team member (OO-T). Once the screening was completed, other team members (OO-T, AT, and MK) reviewed and confirmed the included and excluded studies. Any disagreement was discussed and resolved among all 4 team members at any of these stages. The following data were extracted from 19 (3.1%) of the 611 articles: country, study design, sample size, authors, year of publication, age, sex of the sample, type of MMPU scales, intervention, effect in reducing mobile phone use and MMPU, and key findings. This review used the Quality Assessment Tool for Quantitative Studies developed by the Effective Public Health Practice Project (EPHPP) to evaluate the methodological quality of the eligible studies [24]. Given the small number of papers and the diversity of the apps, a meta-analysis could not be conducted.

#### Content Analysis

Content analysis was used to identify the features of the mobile phone apps (n=13)—designed to reduce mobile phone use and prevent MMPU—that were included in the study. Content analysis is a method used to draw inferences from qualitative data by systematically characterizing themes. This method has frequently been used to analyze apps aimed at reducing mobile phone use while driving [22], minimizing alcohol and illicit substance use [25], aiding smoking cessation [26], monitoring alcohol use [27], and treating depression [28]. The content analysis process comprised two steps: (1) identifying and (2) analyzing features that contribute to reducing mobile phone use and preventing MMPU.

A coding process, based on previous research on similar apps [13,21], was developed to extract key features from the apps. This study adapted the app clustering strategy framework developed in previous literature [13,21] because the initial approaches were found to be incomplete and included distractions related to other devices such as laptop computers as well as the internet. In addition, the aim of the study was to identify how categorization evolved over time based on the reviewed apps, resulting in the creation of new categories. The classification framework used to characterize the key features is presented in Table 1. The first author extracted data from full descriptions, screenshots, videos on the Google Play Store, Apple App Store, app website, and previous review studies. Moreover, the first author also installed each app and examined its functionality for a minimum duration of 30 minutes. Subsequently, the last author checked the data extracted to reach a consensus.

**Table 1:** The classification framework of app features.

Category and feature		Definition	Source of definition
**Self-tracking**			
	Use history and visualization	Provides a summary of mobile phone use history and a visualization (ie, mobile phone use history, notification, most used app, and unlocked count) daily, weekly, or monthly	Monge Roffarello and de Russis [13], Lyngs et al [21], and the authors of this paper
**Social tracking**			
	Social sharing of mobile phone use	Shares progress	Lyngs et al [21]
	Global comparison	Provides information about the global use average and compares it with the user’s mobile phone use	The authors of this paper
**Goal setting**			
	Set goal or group goal	Allows users to set limits on mobile phone use or desired focus time. In addition, users can create groups within the app to set use limits for specific apps, such as social media apps	Lyngs et al [21] and the authors of this paper
	Set activity or interest goal	Allows users to set goals for hobbies or activities	Lyngs et al [21]
	Goal warning	Sends use alert (a reminder when the set limit is approaching)	The authors of this paper
**Blocking**			
	Spontaneous	Allows users to lock the mobile phone quickly (duration can be chosen), lock the mobile phone (until the user chooses to unlock it), or immediately disconnect via 1-click access to the widget	The authors of this paper
	Scheduled	Schedules or sets rules to silence notifications, alerts, and calls; schedules blocklist; schedules off time; disconnects from distracting apps; automatically enters “do not disturb” mode; and automatically locks apps (a password or PIN^a^ must be entered to continue accessing apps, modify the active app block profile, or uninstall the app used to reduce mobile phone use and MMPU^b^)	The authors of this paper
**Gamification**			
	Gain or loss points	Incorporates engaging features to motivate users to reduce mobile phone use. One such feature is the points system, where users can earn rewards by staying focused and lose points for excessive mobile phone use	Lyngs et al [21]
	Real-world reward	Plants trees (ie, *Forest* app)	Lyngs et al [21]
Simplification		Changes the interface display from color to black and white to make for a less engaging screen (ie, grayscale mode); other authors call it minimization [21]	The authors of this paper
Assessment		Assesses the level of addiction to help users understand their current situation	The authors of this paper

^a^PIN: personal identification number.

^b^MMPU: maladaptive mobile phone use.

#### User Reviews

User reviews of 12 (92%) of the 13 apps (the *Screen Time* [iOS] app was excluded because it did not have user reviews) were analyzed using the Appbot platform, which is designed to analyze all reviews for apps [29] and it has been used by Kaur and Chakravarty [30] and Bano et al [31]. User reviews posted in Google Play Store or Apple App Store in English from July 2008 to December 2022 were used to examine users’ perceptions and sentiments regarding the various features of the identified apps. To enhance the depth of the analysis, it was further broken down based on the app features and several custom topics. In this study, sentiment analysis of reviews refers to extracting magnitude (eg, positive, negative, neutral, or mixed) and subjectivity from the text review. Sentiment scores and the cutoff created by Appbot range from 0 to 100, based on the volume of reviews for the period; the trend in review volume (whether it is trending up or down); the trend in the star rating; the ratio of positive, neutral, and negative reviews; and the trend in the percentage of positive, neutral, and negative reviews. More information on calculating the score is available on the Appbot website [29]. The process of extracting the user reviews through the custom topics automatically tracks reviews containing keywords and phrases developed using a Boolean strategy. The results provide insights into how satisfied or dissatisfied users are with a particular feature. All user reviews that contain text from app stores were used to examine users’ perceptions and sentiments regarding the various features of the identified apps. We followed procedures similar to those used sentiment score to assess the satisfaction level of mobile apps for library use [30] and to evaluate the mobile pedagogical affordances of apps [31].

## Results

### Effectiveness of Apps

A total of 19 studies in the literature have evaluated the effectiveness of 13 apps in reducing mobile phone use and MMPU (See Multimedia Appendix 4 for more details). These studies used various types of research designs, including randomized controlled trials (5/19, 26%) [32-36], experimental designs (6/19, 32%) [37-42], interviews (4/19, 21%) [37,40,43,44], cross-sectional designs (8/19, 34%) [11,38,39,44-48], longitudinal studies (3/19, 16%) [34,38,42], and observational research-secondary data [49]. Some of the studies (5/19, 26%) used a combination of different designs [11,37,39,40,44]. The duration of app use in most studies ranged from 3 days to 16 weeks. However, because of the heterogeneity across the studies, including differences in intervention strategies, durations of interventions, methodologies, scales, and study designs, a direct comparison of the 19 studies was not feasible.

To assess the quality of the quantitative studies, the EPHPP guideline was used [24]. The EPHPP guideline consists of 8 criteria: study design, selection bias, dropouts, blinding, intervention integrity (if applicable), data collection, analysis, and confounding. The findings revealed that, of the 19 studies, 1 (5%) was of methodologically strong quality [34], 6 (32%) were of moderate quality [32,33,35,36,39,40], and the remaining 11 (58%) were of weak quality [11,37,38,41,42,44-49]. Meta-analyses of the quantitative data could not be conducted owing to the heterogeneity of the data and the quality of the included studies. Therefore, the effectiveness of app features in reducing mobile phone use and MMPU were summarized narratively, and caution should be exercised when interpreting the findings.

The study findings revealed that 4 (31%) of the 13 apps can effectively reduce mobile phone use. Specifically, *Screen Time* (iOS) [11,32-34,39,40], *Forest* and *Screen Time* [43], and *AntiSocial* [36] were found to be effective in reducing mobile phone use. Various intervention strategies were also found to be effective in reducing mobile phone use, including the use of grayscale mode [11,32,33,39,40], app limit feature [34]; and mixed interventions [11,36]. The use of grayscale mode was found to be particularly effective in reducing mobile phone use, with average reductions of up to 37.90 minutes per day (preintervention: mean 255.34, SD 100.46; postintervention: mean 217.44, SD 94.90; *P*≤.001) [33,39]. Mixed interventions have been shown to be effective; for instance, a study successfully reduced mobile phone use by combining interventions such as self-monitoring mobile phone use (*AntiSocial* app), mindfulness (*Headspace* app), and mood tracking (*Pacifica* app; preintervention: mean 4.515, SD 2.28; postintervention: mean 3.51, SD 1.88; *P*<.001) [36]. Some studies used the evaluated apps without explicitly mentioning their features. An app limit intervention strategy was found to significantly decrease mobile phone use by 6.2% per day (*P*<.05) and Facebook use by 33.2% in the short term (*P*<.01) [34]. In the long term, this strategy can significantly decrease Facebook use by 36.8% (*P*<.001) and Instagram use by 33.9% (*P*<.01) but not substantially reduce mobile phone use [34]. However, although *Forest* and *Screen Time* were found to be effective in reducing mobile phone use [43], the small sample size of the study (n=26) limits the conclusiveness of the results.

Of the 19 papers, 6 (32%) investigated the effect of using apps on MMPU [32,35,36,39-41]. The scales used to measure MMPU were the Mobile Phone Problematic Usage Scale developed by Bianchi and Phillips [50] and used by Ochs and Sauer [32]; Mobile Phone Problem Use Scale developed by Foerster et al [51] and used by Keller et al [35]; Smartphone Addiction Scale Short Version developed by Kwon et al [52] and used by Holte et al [39], Olson et al [40], and Loid et al [41]; Fear of Missing Out Scale developed by Przybylski et al [53] and used by Throuvala et al [36], Nomophobia Questionnaire developed by Yildirim and Correia [54] and used by Throuvala et al [36]; and 4 questions based on the study by Roberts et al [55] and used by Schmuck [47]. The findings were mixed: of the 19 studies, 3 (16%) suggested that apps can decrease MMPU [36,39,40], 3 (16%) suggested that apps do not affect MMPU [35,36,41], and 1 (5%) suggested that apps can even increase MMPU [32]. According to a study, participants who initially scored 35.29 (SD 8.84) on the Smartphone Addiction Scale achieved a lower score of 28.08 (SD 9) after receiving a nudge intervention for 2 to 8 weeks (*P*<.001) [40]. In another study, participants were able to reduce their MMPU score from 27.6 (SD 6.12) to 25 (SD 7.1; *P*<.001) by changing their mobile phone display to grayscale mode for a minimum of 2 weeks [39]. A further study found a significant decrease in mobile phone use per day and in terms of FoMO (preintervention: mean 77.17, SD 2.40; postintervention: mean 78.03, SD 2.72; *P*<.001), but no significant results were observed in terms of nomophobia [36].

### Features of Apps Used to Reduce Mobile Phone Use and MMPU

Features of the 13 apps with scientific evidence about their effectiveness were extracted and categorized (Table 2). The most common features are self-tracking and goal setting, available in all included apps to reduce mobile phone use and MMPU. Social tracking features such as inviting friends or family to join off time together (ie, *Offtime* and *Flipd*), inviting friends or family to share focus mode, tree planting together (ie, *Forest*), or creating a group to share progress and feel accountable to the group (ie, *Space*) are only offered by 4 (31%) of the 13 apps. The assessment feature could help users to understand their mobile phone use and MMPU (ie, *Space*). The only app that provides a gamification method is *Forest,* where, if users stay focused on the task, a digital seed will grow into a tree, and it can be substituted for a real tree in the real world. The simplification offered by 2 (15%) of the 13 apps is a grayscale feature enabled by using the *Screen Time* (iOS) feature within the Settings app and part of the wind down (previously called bedtime mode) in *Digital Wellbeing*.

**Table 2:** Distribution of features.

Name of the app	Self-tracking	Goal setting				Blocking			Gamification			Social tracking			Simplification		Assessment
	Use history and visualization	Set goal or group goal	Set activity or interest goal	Goal warning	Spontaneous		Scheduled	Gain or loss points or grow tree		Real-world reward	Social sharing of mobile phone use		Global comparison				
*AntiSocial*	✓	✓	✓	✓	✓		✓						✓				
*AppDetox*	✓	✓	✓	✓													
*App* *Usage*	✓	✓		✓													
*Detox: Procrastination Blocker*	✓	✓			✓												
*Digital Wellbeing*	✓	✓		✓			✓							✓			
*Flipd*	✓	✓	✓	✓							✓		✓				
*Forest*	✓	✓						✓		✓	✓		✓				
*Screen Time (iOS)*	✓	✓		✓	✓		✓							✓			
*Offtime*	✓	✓	✓		✓		✓										
*QualityTime*	✓	✓	✓	✓	✓		✓										
*RescueTime*	✓	✓	✓	✓	✓		✓										
*Screen Time*	✓	✓															
*Space*	✓	✓									✓		✓			✓	

### User Reviews and Sentiment Analysis

The apps included for in-depth review had 353,043 reviews in all languages, of which 145,454 (41.2%) were in English. *Forest* was the most reviewed app (72,499/145,454, 49.84%) and had the highest positive sentiment (53,649/72,499, 74%), whereas the least reviewed app and the one with the lowest positive sentiment was *RescueTime*. The majority of the user reviews had given 5 stars (87,272/145,454, 60%) to this group of 12 apps (the *Screen Time* [iOS] was excluded because it did not have user reviews), whereas the rest were given 4 stars (17,454/145,454, 12%), 3 stars (10,181/145,454, 7%), 2 stars (5818/145,454, 4%), and 1 star (24,727/145,454, 17%). The sentiment scores ranged from 61 to 86 out of 100, meaning that users tended to express a positive sentiment toward all apps. For details, refer to Table 3 for overall sentiment breakdown of the apps and Table 4 for sentiment breakdown of the app features.

In-depth analyses were conducted on critical themes of the reviews. Table 4 shows the sentiment breakdown of app features. We developed initial relevant search terms through custom topics (Multimedia Appendix 5), whereas machine learning developed by the Appbot software provided the rest. Overall, 21.81% (31,729/145,454) of the reviews reported reduced mobile phone use, 63% (19,989/31,729) of the users had a positive sentiment, and 48% (5505/11,468) users had a positive sentiment toward gamification offered by *Forest*. Users also thought that customer support was helpful (3118/4585, 68%). In addition, users used the apps to help them in some use cases (ie, studying, 3823/4498, 84.99% positive sentiment in reviews; working, 3535/6669, 53.01% positive sentiment in reviews; and sleeping, 120/236, 50.8% positive sentiment in reviews). The first review of a grayscale feature that *Digital Wellbeing* launched appeared in September 2018. Among the reviews about this feature, 56% (200/357) had negative sentiments. Users felt dissatisfied (gave more negative comments) in terms of bugs (7297/32,274, 22.61%), updates (2837/32,274, 8.79%), performance (1415/32,274, 4.38%), notifications and alerts (1350/32,274, 4.18%), too many advertisements (211/32,274, 0.65%), payment (718/32,274, 2.22%), privacy (428/32,274, 1.33%), and battery drain issue (132/32,274, 0.41%).

**Table 3:** Overall sentiment breakdown of apps (N=145,454 reviews). Percentage values correspond to the total number of the reviews for each app.

Name of the app	Sentiment score	Reviews, n	Average rating	Star breakdown						Sentiment breakdown			
				5, n (%)	4, n (%)	3, n (%)	2, n (%)	1, n (%)	Positive, n (%)		Neutral, n (%)	Mixed, n (%)	Negative, n (%)
*Forest*	86	72,499	4.4	49,227 (67.9)	10,418 (14.37)	4596 (6.34)	2762 (3.81)	5495 (7.58)	53,649 (74)		4349 (6)	4349 (6)	10,149 (14)
*Detox: Procrastination Blocker*	83	1362	4.1	870 (63.88)	171 (12.56)	108 (7.93)	58 (4.25)	155 (11.38)	885 (65)		109 (8)	123 (8)	245 (18)
*App* *Usage*	82	1995	4.1	1251 (62.71)	270 (13.53)	150 (7.52)	91 (4.56)	233 (11.68)	1137 (57)		239 (12)	239 (12)	379 (19)
*Space*	79	4262	4.1	2062 (48.38)	1065 (24.99)	431 (10.11)	302 (7.09)	402 (9.43)	2515 (59)		469 (11)	384 (9)	895 (21)
*Screen Time*	78	1868	3.8	1097 (58.73)	230 (12.31)	123 (6.58)	79 (4.23)	339 (18.15)	1233 (66)		112 (6)	112 (6)	411 (22)
*AntiSocial*	73	1236	3.7	550 (44.53)	200 (16.2)	179 (14.5)	116 (9.4)	190 (15.37)	593 (48)		111 (9)	99 (8)	433 (35)
*Digital Wellbeing*	71	50,895	3.3	27,493 (54.02)	3985 (7.83)	2591 (5.09)	1598 (3.14)	15,228 (29.92)	28,501 (56)		3563 (7)	3563 (7)	15,269 (30)
*AppDetox*	70	1303	3.5	448 (34.37)	278 (21.34)	225 (17.27)	149 (11.44)	203 (15.58)	469 (36)		182 (14)	156 (12)	495 (38)
*Offtime*	69	3780	3.4	1568 (41.48)	575 (15.21)	420 (11.11)	353 (9.34)	864 (22.86)	1550 (41)		378 (10)	378 (10)	1474 (39)
*QualityTime*	69	3469	3.5	1400 (40.36)	575 (16.58)	436 (12.57)	363 (10.46)	695 (20.03)	1457 (42)		347 (10)	347 (10)	1318 (38)
*Flipd*	65	2192	3.3	830 (37.85)	318 (14.5)	247 (11.27)	203 (9.28)	594 (27.10)	921 (42)		197 (9)	153 (7)	921 (42)
*RescueTime*	61	593	3.1	180 (30.35)	85 (14.33)	105 (17.71)	75 (12.65)	148 (24.96)	196 (33)		59 (10)	53 (9)	285 (48)

**Table 4:** Sentiment breakdown of app features. Percentage values correspond to the total number of the reviews for each app.

Custom topic		Total reviews (N=145,454)	Sentiment breakdown			
		Reviews, n (%)	Positive, n (%)	Neutral, n (%)	Mixed, n (%)	Negative, n (%)
**Analysis provided by the authors**						
	Reduce mobile phone use and MMPU^a^	31,729 (21.81)	19,989 (63)	2856 (9)	2221 (7)	6663 (22)
	Gamification (only in *Forest*)	11,468 (7.88)	5505 (48)	1606 (14)	1147 (10)	3211 (28)
	Grayscale mode (part of wind down feature in *Digital Wellbeing*)	357 (0.25)	68 (19)	46 (13)	43 (12)	200 (56)
	Blocking	9710 (6.68)	5923 (60)	971 (10)	680 (7)	2136 (22)
	Goal setting	7546 (5.19)	5735 (75)	679 (9)	302 (4)	830 (11)
	Tracking	7819 (5.38)	2815 (35)	1056 (13)	938 (12)	3049 (39)
**Analysis provided by the software**						
	Design and UX^b^	15,565 (10.7)	8872 (57)	1557 (10)	1090 (7)	4047 (26)
	Use cases	9914 (6.82)	6940 (71)	991 (10)	496 (5)	1487 (15)
	Customer support	4585 (3.15)	2613 (58)	459 (10)	459 (10)	1055 (23)
	Feature request	6708 (4.61)	2616 (39)	1207 (18)	805 (12)	2079 (31)
	Notification and alerts	2878 (1.98)	432 (16)	403 (14)	489 (17)	1554 (54)
	Advertising	1517 (1.04)	349 (23)	152 (10)	228 (15)	789 (52)
	Privacy	631 (0.43)	38 (6)	38 (6)	44 (7)	511 (81)
	Battery	231 (0.16)	39 (16)	16 (7)	23 (10)	152 (66)

^a^MMPU: maladaptive mobile phone use.

^b^UX: user experience.

## Discussion

### Principal Findings

Previous research has indicated that there is a lack of scientific evidence regarding the efficacy and effectiveness of apps in delivering health interventions, including apps aimed at reducing mobile phone use [8,56]. As a result, this study aimed to examine the evidence supporting the effectiveness of apps in reducing mobile phone use and addressing MMPU. To the best of our knowledge, no previous study has evaluated such apps and their relationship to different types of MMPU. The findings of this study revealed that various types of MMPU were addressed in 6 (32%) of the 19 papers [32,35,36,39-41], which included the use of the Mobile Phone Problematic Usage Scale developed by Bianchi and Phillips [50] and used by Ochs and Sauer [32]; Mobile Phone Problem Use Scale developed by Foerster et al [51] and used by Keller et al [35]; Smartphone Addiction Scale Short Version developed by Kwon et al [52] and used by Holte et al [39], Olson et al [40], and Loid et al [41]; Fear of Missing Out Scale developed by Przybylski et al [53] and used by Throuvala et al [36]; Nomophobia Questionnaire developed by Yildirim et al [54] and used by Throuvala et al [36]; and an adapted set of 4 questions based on the study of Roberts et al [55] and used by Schmuck [47].

As per existing primary evidence, the apps that have shown effectiveness in reducing mobile phone use and MMPU include *Screen Time* (iOS; with moderate to strong evidence) [11,32-34,39,40], *AntiSocial* (strong evidence) [36], and *Forest* and *Screen Time* (with weak evidence) [43]. However, it is important to note that 2 (11%) of the 19 studies included a mixed intervention (where using apps was just one of them) to reduce mobile phone use and MMPU [11,36]. Specifically, the grayscale feature offered by *Screen Time* (iOS) has been shown to be effective in reducing mobile phone use [11,32,33,36,39,40]. Some of the studies (6/19, 32%) directly assessed the impact of apps on MMPU. Among the 6 studies that examined MMPU, 3 (50%) demonstrated that the grayscale feature could lower MMPU [36,39,40], whereas 3 (50%) found that apps as an intervention did not reduce MMPU [35,36,41]. Finally, 1 (17%) of these 6 studies yielded contrasting results, indicating an increase in users’ MMPU [32]. A study by [36] showed that mixed intervention could lower FoMO, but not for Nomophobia.

Regarding the features of the apps, we identified 7 categories: self-tracking, social tracking, goal setting, blocking, gamification, simplification, and assessment. The most popular features were self-tracking and goal setting, whereas gamification and assessment were less commonly observed. The most comprehensive apps in terms of features were *AntiSocial*, with *Forest* being the only one offering gamification. *Space* provided an assessment of MMPU by asking users a few short questions, helping them to understand their mobile phone use and MMPU. However, the effectiveness of gamification and assessment in reducing mobile phone use has not been thoroughly examined in the literature. Social tracking features, such as inviting friends and family to join off time together (eg, *Offtime* and *Flipd*), inviting them to share focus mode and tree planting together (eg, *Forest*), or creating a group to share progress and foster accountability (eg, *Space*), were only offered by 4 (31%) of the 13 apps.

The sentiment scores ranged from 61 to 86 out of 100, indicating that users tend to accept apps that address mobile phone use reduction. The highest score was obtained by *Forest*, thanks to its gamification feature that helps users to focus more on their activities. Of note, adoption and sentiment information for *Screen Time* (iOS) were not available because this is a built-in app in the iPhone. Thus, it remains unclear how it has been adopted and received by the public. Analyzing sentiment around features in all apps, we found that users had a negative sentiment associated with the grayscale in *Digital Wellbeing* (200/357, 56%) as part of the wind down feature. This low acceptance suggests that users do not prefer using it, and there might be less adoption of this feature. Future research should explore ways to enhance the acceptability of this feature because some studies have concluded that grayscale mode enabled by using the *Screen Time* feature within the Settings app (iOS) effectively reduces mobile phone use [11,32,33,39,40] and MMPU [39,40].

Reducing mobile phone use and MMPU involves limiting meaningless use and supporting meaningful use [57]. The uses and gratifications theory can be applied to examine how user motivation (specific intention or general habit) and the type of use (eg, information seeking or communication) affect the meaning derived from mobile phone use [58]. At times, mobile phone users may feel a lack of autonomy and meaning, leading them to seek gratification by escaping from the situation [57]. On the basis of an autoethnographic study and functionality review, most apps primarily focus on limiting screen time by creating obstacles to restrict use, promoting awareness of reaching set limits, supporting focused attention, and encouraging motivation to limit use [59]. However, further work is needed to define strategies that provide gratification from limiting mobile phone use.

Although some apps are effective in helping to reduce mobile phone use, without user motivation and commitment, they are likely to fail [36]. Personal characteristics of users, such as an awareness of the relationship between mobile phone use behavior and well-being as well as the motivation to achieve goals, will influence the results [44,60]. Therefore, app developers should allow users to maintain their freedom of choice and provide content that promotes motivation and commitment to facilitate the adoption of new habits and engagement in long-term behavior change [60,61]. Some recommendations from consumers’ point of view include ensuring that the app is enjoyable to use, meets their needs, supports existing habits, allows for goal creation and modification, and provides rewards and opportunities to share progress within a social community. Currently, few apps include reinforcing factors such as social sharing of mobile phone use, which is considered important for long-term adoption of behavior change [61].

In future research, it is important to address certain limitations. First, the samples in the literature reviews included in this study exhibited cultural and social similarities in terms of research locations, age groups, and educational backgrounds. However, for a more comprehensive understanding of our diverse world, it is crucial to incorporate a heterogeneous sample that encompasses a broader range of cultural, social, and demographic characteristics. Different age groups may exhibit varying texting and mobile phone etiquette norms [62], and mobile phone use tends to decline linearly with age, whereas MMPU remains relatively stable throughout adulthood before declining rapidly around the age of 40 years [63]. In addition, the samples in the included studies predominantly consisted of individuals with a high level of education, which can influence technology perceptions and use patterns. It is worth noting that users in these studies may have been more familiar with technology than the general population. Previous research has indicated that young people with higher education, excellent health, and higher income tend to be the primary users of health apps [64]. Second, it is possible that we missed certain apps that were being trialed and had not yet been published because we only included active apps that had undergone scientific evaluation. Third, it is important to acknowledge that user reviews are not the sole method for conducting sentiment analysis. Alternative approaches should also be considered. Fourth, future research should not only explore reduction in mobile phone use but also focus on meaningful use as a better approach to addressing and decreasing MMPU. Fifth and last, the heterogeneity of intervention strategies, duration, scale, and methods used in the included studies prevented us from conducting a meta-analysis. Future research should consider these limitations and strive to address them effectively.

### Conclusions

This paper investigated existing apps designed to reduce mobile phone use and prevent MMPU and examines the evidence of their effectiveness. To the best of our knowledge, no previous study has conducted a comprehensive review and sentiment analysis of apps aimed at reducing mobile phone use and MMPU. Although our study demonstrates the effectiveness of app-based management as a strategy for reducing mobile phone use and MMPU, further research is necessary to evaluate the efficacy of app-based interventions. According to the scientific literature, *Screen Time* (iOS) and *AntiSocial* showed moderate to strong evidence, whereas *Forest* and *Screen Time* exhibited a weak level of evidence, in reducing mobile phone use and MMPU. Effective intervention strategies for reducing mobile phone use and MMPU included using grayscale mode, app limit features, and mixed interventions. In addition, self-tracking and goal setting were found to be the most popular features, whereas gamification and assessment were less frequently used. Users generally displayed positive sentiment toward these apps, with sentiment scores ranging from 61 to 86 out of 100. Notably, *Forest* received the highest score owing to its gamification feature, which aids users in enhancing focus on their activities. This study holds significance because the public’s use of apps to reduce mobile phone use and MMPU is likely to increase. Therefore, users should be better informed about the factors that make an app appealing and successful in facilitating behavior change. Collaboration among researchers, app developers, mobile phone manufacturers, and policy makers can contribute to the delivery and evaluation of apps aimed at reducing mobile phone use and MMPU.

## Appendix

Multimedia Appendix 1 Characteristics of the reviewed apps.

Multimedia Appendix 2 Search strategy.

Multimedia Appendix 3 PRISMA (Preferred Reporting Items for Systematic Reviews and Meta-Analyses) checklist.

Multimedia Appendix 4 Summary of published literature evaluating the effectiveness of apps.

Multimedia Appendix 5 Search terms in the custom topics for user reviews.

## Abbreviations

EPHPP: Effective Public Health Practice Project
FoMO: fear of missing out
MMPU: maladaptive mobile phone use
PRISMA: Preferred Reporting Items for Systematic Reviews and Meta-Analyses
RQ: research question

## Data Availability

The data set used to conduct sentiment analysis is publicly available and can be accessed over the internet [65].
