# Supplementary material for: Evaluating the Effectiveness of Apps Designed to Reduce Mobile Phone Use and Prevent Maladaptive Mobile Phone Use: Multimethod Study
Source: J Med Internet Res. 2023 Aug 29;25:e42541. doi: 10.2196/42541 (PMC10498313; doi:10.2196/42541)
Supplement: Multimedia Appendix 1 [file jmir_v25i1e42541_app1.docx]

#### **Multimedia Appendix 1.** Characteristics of the reviewed apps (Accessed on 28^th^ February 2023)

| **No** | **App** | **Description** | **Last Update** | **Installs** | **User Review** | **Platform** | **Access** |
| --- | --- | --- | --- | --- | --- | --- | --- |
| **Included apps** | | | | | | | |
| 1 | *QualityTime* | A phone addiction manager to help find digital habits and reduce phone usage by scheduling breaktime (e.g., study time, family time). | 3 Feb 2023 | 1,000,000+ | 20,500 | Android | Freemium |
| 2 | *Detox Proc. Blocker* | A focusing app for recovering procrastinators breaks free from needless smartphone use, boosts self-control, and stays focused with a digital detox. | 26 Jun 2022 | 1,000,000+ | 17,200 | Android | Freemium |
| 3 | *OFFTIME* | An app that can help to monitor and customise connectivity | 9 Nov 2020 | 1,000,000+ | 18,132 | Android | Freemium |
| 4 | *SPACE* | An app to understand addiction type, manage phone addiction, help restrict mobile usage, and change habits with friends and family. | 19 Jan 2023 | 1,000,000+ | 28,800 | Android | Freemium |
| 5 | *Forest* | A gamification app that helps to eliminate distractions, stay focused, and build commitment while growing trees as a representation of the efforts. | 16 Feb 2023 | 10,000,000+ | 567,000 | Android | Freemium |
| 6 | *AppDetox* | An app that helps to get more social time by setting rules for the apps and notifications and reminding people to take a break and stop their heavy *App Usage*. | 21 Jun 2020 | 100,000+ | 3,300 | Android | Freemium |
| 7 | *ActionDash* | A screen time helper and self-control to reduce screen time and stay | 18 Feb 2023 | 1,000,000+ | 56,000 | Android | Freemium |
| 8 | *RescueTime* | A productivity and time tracker helps understand and control phone usage, build better habits, and beat distractions. | 18 Nov 2021 | 500,000+ | 3,170 | Android | Freemium |
| 9 | *Digitox* | An app that helps to improve *Digital Wellbeing* by understanding digital habits, setting digital app limits, and reminding wisely when the time limit is exceeded. | 7 Sept 2022 | 500,000+ | 8,720 | Android | Freemium |
| 10 | *Digital Wellbeing* | A built-in app which helps to understand digital habits, set the goal, and disconnect to selected app. | 24 Jan 2023 | Built-in app | 795,000 | Android | Free |
| 11 | *iOS Screen Time* | A built-in app on iPhone 12 or up to help people more aware of their phone usage. | - | Built-in app | - | iOS | Free |
| 12 | *Flipd* | An app that helps to focus on study goals, track progress and improve over time. | 23 Feb 2023 | 1,000,000+ | 5,660 | Android | Freemium |
| 13 | *AntiSocial* | An app that helps to focus and minimise distractions by offering tools to manage, block, and control phone usage. | 31 Oct 2020 | 500,000+ | 8,310 | Android | Freemium |
|  | | | | | | | |
| **Excluded apps** | | | | | | | |
| 1 | Social Fever | Beat smartphone addiction by managing time wisely and balancing digital and real life. | 1 Mar 2022 | 100,000+ | 381 | Android | Freemium |
| 2 | *App Usage* | Device usage management app | 23 Feb 2023 | 1,000,000+ | 12,600 | Android | Freemium |
| 3 | *My Addictometer* | It is a productivity tool to save and utilise time by staying away from their smartphone. | 24 Sep 2018 | 100,000+ | 416 | Android | Freemium |
| 4 | *AppBlock* | A mobile tracker manager helps block annoying apps, track screen time, and set goals and focus. | 15 Feb 2023 | 1,000,000+ | 99,100 | Android | Freemium |
| 5 | *Stay Focused* | An app that helps to focus by restricting the daily usage of blocked apps to the selected time. | 6 Feb 2023 | 1,000,000+ | 65,000 | Android | Freemium |
| 6 | *Ubhind* | A mobile tracker manager that helps to control phone usage by automatically locking if used for longer than the set time. | 19 Jan 2023 | 1,000,000+ | 38,100 | Android | Freemium |
| 7 | *Keep Me Out* | An app that helps to stay focused and enjoy distraction-free time by locking the device, but we still have access to the lock screen and its widget to make emergency calls and answer calls. | 8 Feb 2022 | 500,000+ | 7,310 | Android | Freemium |
| 8 | *Freedom* | A blocker app helps to stay focused, productive, and engage with the real world by temporarily blocking time-wasting apps. | 13 Jan 2023 | 500,000+ | 4,270 | Android | Freemium |
| 9 | *Daywise* | A smart box which helps to stay focused by scheduling notifications in batches. | 2 Feb 2021 | 100,000+ | 712 | Android | Freemium |
| 10 | *Actuflow* | An app that offers an intentional way to use a phone by prompting you to write the purpose of usage every time when you unlock your phone. This app challenges our commitment to using a phone for meaningful reasons. | 17 Mar 2021 | 10,000+ | 151 | Android | Freemium |
| 11 | *RealizD* | An app that helps to know phone usage and overcome the addiction by setting up controls and enabling reminders to develop phone habits. Not available | 1 Jun 2019 | 50,000+ | 220 | iOS | Freemium |
| 12 | *Stayfree* | A screen time tracker and limited *App Usage* help to focus and boost productivity. | 20 Feb 2023 | 5,000,000+ | 148,000 | Android | Freemium |
| 13 | *Off the Grid* | A digital detox app that helps curb phone addiction by scheduling off the grid time. | 18 Feb 2022 | 100,000+ | 1,390 | Android | Freemium |
| 14 | *FocusMe* | An app that helps to break bad usage habits by blocking or setting time app limits. | 31 Aug 2020 | 50,000+ | 650 | Android | Freemium |
| 15 | *Menthal* | An app for digital dieting and a sustainable digital lifestyle helps fight problematic phone use, shows how our mood develops with a food diary, and finds our personality traits. | 6 Sept 2022 | 500,000+ | 7,590 | Android | Freemium |
| 16 | *SocialX* | An app which helps track and reduce screen time on social media apps. | 28 Dec 2022 | 50.000+ | 3,520 | Android | Freemium |
| 17 | *My Phone Time* | An *App Usage* tracking helps to limit phone time and boost focus and productivity. | 12 Oct 2020 | 100.000+ | 3,580 | Android | Free |
| 18 | *Minimalist* *Phone* | An app which helps to reduce distraction by turning the screen phone into a minimalist interface. | 31 Jan 2023 | 500.000+ | 8,420 | Android | Freemium |
| 19 | *FOMO* | An app which helps to beat phone addiction and go from Fear of Missing Out to JOMO the joy of missing out by measuring FOMO level. | 30 Sept 2022 | 100.000+ | 132 | Android | Freemium |
| 20 | *Lock Me Out* | An app that helps to reduce phone usage by blocking selected apps and scheduling lockouts. | 13 Feb 2023 | 500.000+ | 7,260 | Android | Freemium |
| 21 | *Focus Plant* | A gamification app, a focus timer, and a study timer help beat phone addiction, and improve productivity, self-control, and concentration. | 12 Feb 2023 | 1.000.000+ | 29,300 | Android | Freemium |
| 22 | *Iron Will* | A simple app that puts a widget on the home screen of the phone so that we can constantly see the progress and keep motivated during the journey by the quotes provided. | 23 Feb 2020 | 500.000+ | 20,900 | Android | Freemium |
| 23 | *Boring Phone* | A launcher that helps save time and reduce phone usage by letting us use only 4 or 8 apps, take control of the colours and the app appearance. | 28 Jan 2020 | 10.000+ | 111 | Android | Freemium |
| 24 | *Focus Quest* | A productivity RPG gamification that helps beat phone addiction and manage concentration. | 23 Feb 2023 | 500.000+ | 2,920 | Android | Freemium |
| 25 | *Apprison* | An app that helps to reduce distraction by creating a prison for all the apps that distract users. | 27 Oct 2021 | 5.000+ | 30 | Android | Freemium |
| 26 | Dopamine Detox | An app that helps change bad habits and reduce phone use. | 24 Mar 2021 | 10.000+ | 784 | Android | Freemium |
| 27 | DTox | An app that helps to break free from procrastination and distractions, boost productivity, and stay focused by disconnecting to the phone for certain hours. | 9 Feb 2023 | 50,000+ | NA | Android | Freemium |
| 28 | *Stay Blocked* | A control app that helps to control phone usage by setting specific times and dates. | 8 Jul 2021 | 5.000+ | 177 | Android | Freemium |
| 29 | *YourHour* | A phone addiction tracker and controller which helps to achieve digital wellness with variety of fun, user-friendly and personalised feature. | 4 Oct 2022 | 1,000,000+ | 73,200 | Android | Freemium |
| 30 | *HelpMeFocus* | An app which helps to stay focused by scheduling app block. | 22 Jun 2020 | 100.000+ | 4,790 | Android | Freemium |
| 31 | Sma-*Phospital* | An app that can diagnose the addiction level and helps to get healthy smartphone/life balance. | 23 May 2022 | 500.000+ | 2.213 | Android | Freemium |
| 32 | *ScreenZen* | A screen time control which helps build a balanced relationship with a phone by offering shortcuts to allow pause before opening a distracting app. | NA | NA | 181 | iPadOS 14.1 or up | Freemium |
| 33 | *OPAL* | A science-based approach to disconnect distractions from phone, set intentions, schedule time off, and focus on what matters. | 23 Nov 2021 | 100,000+ | 1,140 | iPadOS 13.2 or up | Freemium |
| 34 | *Screen Time* | A time management awareness tool which helps to make users more aware of smartphone habits and adjust accordingly. | 21 Jul 2022 | 1,000,000+ | 34,900 | Android | Freemium |
| 35 | *ActionFree* | Screen Time tracker which helps to limit app usage and overcome phone addiction. | 8 Mar 2021 | 100+ | NA | Android | Freemium |
| 36 | *Offscreen* | An app helps to track phone use and stay focus. | 16 Oct 2022 | 100,000+ | NA | Android | Freemium |
| 37 | *Attention* | An app helps increase awareness by questioning why you open an app and how you spend your time. | 23 Jul 2021 | 1,000+ | NA | Android | Freemium |
| 38 | *Digital distancing* | A tool to fight phone addiction by customising the detox challenge. | 31 Dec 2021 | 500+ | NA | Android | Freemium |
| 39 | *Phonies* | An app to control phone usage by tracking and limiting phone unlocking. | 24 Nov 2019 | 100+ | NA | Android | Freemium |
| 40 | *Overcome Phone Addiction* | An app helps to balance phone use by locking technology. | 22 Jul 2019 | 100+ | NA | Android | Freemium |
| 41 | *Timelimit.io* | A tool to group apps into categories to limit the time | 15 Feb 2023 | 10,000+ | 303 | Android | Freemium |
| 42 | *AddiLock* | An app that helps multitask productively, track phone use, and block app which cause distraction. | 4 Aug 2018 | 500+ | NA | Android | Freemium |
