# Supplementary material for: Evaluating the Effectiveness of Apps Designed to Reduce Mobile Phone Use and Prevent Maladaptive Mobile Phone Use: Multimethod Study
Source: J Med Internet Res. 2023 Aug 29;25:e42541. doi: 10.2196/42541 (PMC10498313; doi:10.2196/42541)
Supplement: Multimedia Appendix 2 [file jmir_v25i1e42541_app2.docx]

#### **Multimedia Appendix 2.** Search strategy

Search strategy: (("name of the potential app" AND “app”) AND ("reduce" OR "limit") AND ("screen time" OR "phone use"))

| **Database** | **No** | **Search terms** | **Results** |
| --- | --- | --- | --- |
| SCOPUS | #1 | TITLE-ABS-KEY(((“QualityTime” AND “app”) AND (“reduce” OR “limit”) AND (“phone use” OR “screen time”))) AND  ( LIMIT-TO ( LANGUAGE ,  "English" ) ) | 51 |
|  | #2 | TITLE-ABS-KEY(((“Detox Proc. Blocker” AND “app”) AND (“reduce” OR “limit”) AND (“phone use” OR “screen time”))) AND  ( LIMIT-TO ( LANGUAGE ,  "English" ) ) |  |
|  | #3 | TITLE-ABS-KEY(((“OFFTIME” AND “app”) AND (“reduce” OR “limit”) AND (“phone use” OR “screen time”))) AND  ( LIMIT-TO ( LANGUAGE ,  "English" ) ) |  |
|  | #4 | TITLE-ABS-KEY(((“SPACE” AND “app”) AND (“reduce” OR “limit”) AND (“phone use” OR “screen time”))) AND  ( LIMIT-TO ( LANGUAGE ,  "English" ) ) |  |
|  | #5 | TITLE-ABS-KEY(((“Forest” AND “app”) AND (“reduce” OR “limit”) AND (“phone use” OR “screen time”))) AND  ( LIMIT-TO ( LANGUAGE ,  "English" ) ) |  |
|  | #6 | TITLE-ABS-KEY(((“AppDetox” AND “app”) AND (“reduce” OR “limit”) AND (“phone use” OR “screen time”))) AND  ( LIMIT-TO ( LANGUAGE ,  "English" ) ) |  |
|  | #7 | TITLE-ABS-KEY(((“ActionDash” AND “app”) AND (“reduce” OR “limit”) AND (“phone use” OR “screen time”))) AND  ( LIMIT-TO ( LANGUAGE ,  "English" ) ) |  |
|  | #8 | TITLE-ABS-KEY(((“RescueTime” AND “app”) AND (“reduce” OR “limit”) AND (“phone use” OR “screen time”))) AND  ( LIMIT-TO ( LANGUAGE ,  "English" ) ) |  |
|  | #9 | TITLE-ABS-KEY(((“Digitox” AND “app”) AND (“reduce” OR “limit”) AND (“phone use” OR “screen time”))) AND  ( LIMIT-TO ( LANGUAGE ,  "English" ) ) |  |
|  | #10 | TITLE-ABS-KEY(((“Digital Wellbeing” AND “app”) AND (“reduce” OR “limit”) AND (“phone use” OR “screen time”))) AND  ( LIMIT-TO ( LANGUAGE ,  "English" ) ) |  |
|  | #11 | TITLE-ABS-KEY(((“iOS Screen Time” AND “app”) AND (“reduce” OR “limit”) AND (“phone use” OR “screen time”))) AND  ( LIMIT-TO ( LANGUAGE ,  "English" ) ) |  |
|  | #12 | TITLE-ABS-KEY(((“Flipd” AND “app”) AND (“reduce” OR “limit”) AND (“phone use” OR “screen time”))) AND  ( LIMIT-TO ( LANGUAGE ,  "English" ) ) |  |
|  | #13 | TITLE-ABS-KEY(((“AntiSocial” AND “app”) AND (“reduce” OR “limit”) AND (“phone use” OR “screen time”))) AND  ( LIMIT-TO ( LANGUAGE ,  "English" ) ) |  |
|  | #14 | TITLE-ABS-KEY(((“Social Fever” AND “app”) AND (“reduce” OR “limit”) AND (“phone use” OR “screen time”))) AND  ( LIMIT-TO ( LANGUAGE ,  "English" ) ) |  |
|  | #15 | TITLE-ABS-KEY(((“App Usage” AND “app”) AND (“reduce” OR “limit”) AND (“phone use” OR “screen time”))) AND  ( LIMIT-TO ( LANGUAGE ,  "English" ) ) |  |
|  | #16 | TITLE-ABS-KEY((My Addictometer” AND “app”) AND (“reduce” OR “limit”) AND (“phone use” OR “screen time”))) AND  ( LIMIT-TO ( LANGUAGE ,  "English" ) ) |  |
|  | #17 | TITLE-ABS-KEY(((“AppBlock” AND “app”) AND (“reduce” OR “limit”) AND (“phone use” OR “screen time”))) AND  ( LIMIT-TO ( LANGUAGE ,  "English" ) ) |  |
|  | #18 | TITLE-ABS-KEY(((“Stay Focused” AND “app”) AND (“reduce” OR “limit”) AND (“phone use” OR “screen time”))) AND  ( LIMIT-TO ( LANGUAGE ,  "English" ) ) |  |
|  | #19 | TITLE-ABS-KEY(((“Ubhind” AND “app”) AND (“reduce” OR “limit”) AND (“phone use” OR “screen time”))) AND  ( LIMIT-TO ( LANGUAGE ,  "English" ) ) |  |
|  | #20 | TITLE-ABS-KEY(((“Keep Me Out” AND “app”) AND (“reduce” OR “limit”) AND (“phone use” OR “screen time”))) AND  ( LIMIT-TO ( LANGUAGE ,  "English" ) ) |  |
|  | #21 | TITLE-ABS-KEY(((“Freedom” AND “app”) AND (“reduce” OR “limit”) AND (“phone use” OR “screen time”))) AND  ( LIMIT-TO ( LANGUAGE ,  "English" ) ) |  |
|  | #22 | TITLE-ABS-KEY(((“Daywise” AND “app”) AND (“reduce” OR “limit”) AND (“phone use” OR “screen time”))) AND  ( LIMIT-TO ( LANGUAGE ,  "English" ) ) |  |
|  | #23 | TITLE-ABS-KEY(((“Actuflow” AND “app”) AND (“reduce” OR “limit”) AND (“phone use” OR “screen time”))) AND  ( LIMIT-TO ( LANGUAGE ,  "English" ) ) |  |
|  | #24 | TITLE-ABS-KEY(((“RealizD” AND “app”) AND (“reduce” OR “limit”) AND (“phone use” OR “screen time”))) AND  ( LIMIT-TO ( LANGUAGE ,  "English" ) ) |  |
|  | #25 | TITLE-ABS-KEY(((“Stayfree” AND “app”) AND (“reduce” OR “limit”) AND (“phone use” OR “screen time”))) AND  ( LIMIT-TO ( LANGUAGE ,  "English" ) ) |  |
|  | #26 | TITLE-ABS-KEY(((“Off the Grid” AND “app”) AND (“reduce” OR “limit”) AND (“phone use” OR “screen time”))) AND  ( LIMIT-TO ( LANGUAGE ,  "English" ) ) |  |
|  | #27 | TITLE-ABS-KEY(((“Focus Me” AND “app”) AND (“reduce” OR “limit”) AND (“phone use” OR “screen time”))) AND  ( LIMIT-TO ( LANGUAGE ,  "English" ) ) |  |
|  | #28 | TITLE-ABS-KEY(((“Menthal” AND “app”) AND (“reduce” OR “limit”) AND (“phone use” OR “screen time”))) AND  ( LIMIT-TO ( LANGUAGE ,  "English" ) ) |  |
|  | #29 | TITLE-ABS-KEY(((“SocialX” AND “app”) AND (“reduce” OR “limit”) AND (“phone use” OR “screen time”))) AND  ( LIMIT-TO ( LANGUAGE ,  "English" ) ) |  |
|  | #30 | TITLE-ABS-KEY(((“My Phone Time” AND “app”) AND (“reduce” OR “limit”) AND (“phone use” OR “screen time”))) AND  ( LIMIT-TO ( LANGUAGE ,  "English" ) ) |  |
|  | #31 | TITLE-ABS-KEY(((“Minimalist Phone” AND “app”) AND (“reduce” OR “limit”) AND (“phone use” OR “screen time”))) AND  ( LIMIT-TO ( LANGUAGE ,  "English" ) ) |  |
|  | #32 | TITLE-ABS-KEY(((“FOMO” AND “app”) AND (“reduce” OR “limit”) AND (“phone use” OR “screen time”))) AND  ( LIMIT-TO ( LANGUAGE ,  "English" ) ) |  |
|  | #33 | TITLE-ABS-KEY(((“Lock Me Out” AND “app”) AND (“reduce” OR “limit”) AND (“phone use” OR “screen time”))) AND  ( LIMIT-TO ( LANGUAGE ,  "English" ) ) |  |
|  | #34 | TITLE-ABS-KEY(((“Focus Plant” AND “app”) AND (“reduce” OR “limit”) AND (“phone use” OR “screen time”))) AND  ( LIMIT-TO ( LANGUAGE ,  "English" ) ) |  |
|  | #35 | TITLE-ABS-KEY(((“Iron Will” AND “app”) AND (“reduce” OR “limit”) AND (“phone use” OR “screen time”))) AND  ( LIMIT-TO ( LANGUAGE ,  "English" ) ) |  |
|  | #36 | TITLE-ABS-KEY(((“Boring Phone” AND “app”) AND (“reduce” OR “limit”) AND (“phone use” OR “screen time”))) AND  ( LIMIT-TO ( LANGUAGE ,  "English" ) ) |  |
|  | #37 | TITLE-ABS-KEY(((“Focus Quest” AND “app”) AND (“reduce” OR “limit”) AND (“phone use” OR “screen time”))) AND  ( LIMIT-TO ( LANGUAGE ,  "English" ) ) |  |
|  | #38 | TITLE-ABS-KEY(((“Apprison” AND “app”) AND (“reduce” OR “limit”) AND (“phone use” OR “screen time”))) AND  ( LIMIT-TO ( LANGUAGE ,  "English" ) ) |  |
|  | #39 | TITLE-ABS-KEY(((“Dopamine Detox” AND “app”) AND (“reduce” OR “limit”) AND (“phone use” OR “screen time”))) AND  ( LIMIT-TO ( LANGUAGE ,  "English" ) ) |  |
|  | #40 | TITLE-ABS-KEY(((“DTox” AND “app”) AND (“reduce” OR “limit”) AND (“phone use” OR “screen time”))) AND  ( LIMIT-TO ( LANGUAGE ,  "English" ) ) |  |
|  | #41 | TITLE-ABS-KEY(((“Stay Blocked” AND “app”) AND (“reduce” OR “limit”) AND (“phone use” OR “screen time”))) AND  ( LIMIT-TO ( LANGUAGE ,  "English" ) ) |  |
|  | #42 | TITLE-ABS-KEY(((“YourHour” AND “app”) AND (“reduce” OR “limit”) AND (“phone use” OR “screen time”))) AND  ( LIMIT-TO ( LANGUAGE ,  "English" ) ) |  |
|  | #43 | TITLE-ABS-KEY(((“HelpMeFocus” AND “app”) AND (“reduce” OR “limit”) AND (“phone use” OR “screen time”))) AND  ( LIMIT-TO ( LANGUAGE ,  "English" ) ) |  |
|  | #44 | TITLE-ABS-KEY(((“Sma-Phospital” AND “app”) AND (“reduce” OR “limit”) AND (“phone use” OR “screen time”))) AND  ( LIMIT-TO ( LANGUAGE ,  "English" ) ) |  |
|  | #45 | TITLE-ABS-KEY(((“ScreenZen” AND “app”) AND (“reduce” OR “limit”) AND (“phone use” OR “screen time”)) AND  ( LIMIT-TO ( LANGUAGE ,  "English" ) ) |  |
|  | #46 | TITLE-ABS-KEY(((“OPAL” AND “app”) AND (“reduce” OR “limit”) AND (“phone use” OR “screen time”))) AND  ( LIMIT-TO ( LANGUAGE ,  "English" ) ) |  |
|  | #47 | TITLE-ABS-KEY(((“Screen Time” AND “app”) AND (“reduce” OR “limit”) AND (“phone use” OR “screen time”))) AND  ( LIMIT-TO ( LANGUAGE ,  "English" ) ) |  |
|  | #48 | TITLE-ABS-KEY(((“ActionFree” AND “app”) AND (“reduce” OR “limit”) AND (“phone use” OR “screen time”))) AND  ( LIMIT-TO ( LANGUAGE ,  "English" ) ) |  |
|  | #49 | TITLE-ABS-KEY(((“Offscreen” AND “app”) AND (“reduce” OR “limit”) AND (“phone use” OR “screen time”))) AND  ( LIMIT-TO ( LANGUAGE ,  "English" ) ) |  |
|  | #50 | TITLE-ABS-KEY(((“Attention” AND “app”) AND (“reduce” OR “limit”) AND (“phone use” OR “screen time”))) AND  ( LIMIT-TO ( LANGUAGE ,  "English" ) ) |  |
|  | #51 | TITLE-ABS-KEY(((“Digital distancing” AND “app”) AND (“reduce” OR “limit”) AND (“phone use” OR “screen time”))) AND  ( LIMIT-TO ( LANGUAGE ,  "English" ) ) |  |
|  | #52 | TITLE-ABS-KEY(((“Phonies” AND “app”) AND (“reduce” OR “limit”) AND (“phone use” OR “screen time”))) AND  ( LIMIT-TO ( LANGUAGE ,  "English" ) ) |  |
|  | #53 | TITLE-ABS-KEY(((“Overcome Phone Addiction” AND “app”) AND (“reduce” OR “limit”) AND (“phone use” OR “screen time”))) AND  ( LIMIT-TO ( LANGUAGE ,  "English" ) ) |  |
|  | #54 | TITLE-ABS-KEY(((“Timelimit.io” AND “app”) AND (“reduce” OR “limit”) AND (“phone use” OR “screen time”))) AND  ( LIMIT-TO ( LANGUAGE ,  "English" ) ) |  |
|  | #55 | TITLE-ABS-KEY(((“AddiLock” AND “app”) AND (“reduce” OR “limit”) AND (“phone use” OR “screen time”))) AND  ( LIMIT-TO ( LANGUAGE ,  "English" ) ) |  |
| PUBMED | #1 | ((“QualityTime” AND “app”) AND (“reduce” OR “limit”) AND (“phone use” OR “screen time”)) | 101 |
|  | #2 | ((“Detox Proc. Blocker” AND “app”) AND (“reduce” OR “limit”) AND (“phone use” OR “screen time”)) |  |
|  | #3 | ((“OFFTIME” AND “app”) AND (“reduce” OR “limit”) AND (“phone use” OR “screen time”)) |  |
|  | #4 | ((“SPACE” AND “app”) AND (“reduce” OR “limit”) AND (“phone use” OR “screen time”)) |  |
|  | #5 | ((“Forest” AND “app”) AND (“reduce” OR “limit”) AND (“phone use” OR “screen time”)) |  |
|  | #6 | ((“AppDetox” AND “app”) AND (“reduce” OR “limit”) AND (“phone use” OR “screen time”)) |  |
|  | #7 | ((“ActionDash” AND “app”) AND (“reduce” OR “limit”) AND (“phone use” OR “screen time”)) |  |
|  | #8 | ((“RescueTime” AND “app”) AND (“reduce” OR “limit”) AND (“phone use” OR “screen time”)) |  |
|  | #9 | ((“Digitox” AND “app”) AND (“reduce” OR “limit”) AND (“phone use” OR “screen time”)) |  |
|  | #10 | ((“Digital Wellbeing” AND “app”) AND (“reduce” OR “limit”) AND (“phone use” OR “screen time”)) |  |
|  | #11 | ((“iOS Screen Time” AND “app”) AND (“reduce” OR “limit”) AND (“phone use” OR “screen time”)) |  |
|  | #12 | ((“Flipd” AND “app”) AND (“reduce” OR “limit”) AND (“phone use” OR “screen time”)) |  |
|  | #13 | ((“AntiSocial” AND “app”) AND (“reduce” OR “limit”) AND (“phone use” OR “screen time”)) |  |
|  | #14 | ((“Social Fever” AND “app”) AND (“reduce” OR “limit”) AND (“phone use” OR “screen time”)) |  |
|  | #15 | ((“App Usage” AND “app”) AND (“reduce” OR “limit”) AND (“phone use” OR “screen time”)) |  |
|  | #16 | (My Addictometer” AND “app”) AND (“reduce” OR “limit”) AND (“phone use” OR “screen time”)) |  |
|  | #17 | ((“AppBlock” AND “app”) AND (“reduce” OR “limit”) AND (“phone use” OR “screen time”)) |  |
|  | #18 | ((“Stay Focused” AND “app”) AND (“reduce” OR “limit”) AND (“phone use” OR “screen time”)) |  |
|  | #19 | ((“Ubhind” AND “app”) AND (“reduce” OR “limit”) AND (“phone use” OR “screen time”)) |  |
|  | #20 | ((“Keep Me Out” AND “app”) AND (“reduce” OR “limit”) AND (“phone use” OR “screen time”)) |  |
|  | #21 | ((“Freedom” AND “app”) AND (“reduce” OR “limit”) AND (“phone use” OR “screen time”)) |  |
|  | #22 | ((“Daywise” AND “app”) AND (“reduce” OR “limit”) AND (“phone use” OR “screen time”)) |  |
|  | #23 | ((“Actuflow” AND “app”) AND (“reduce” OR “limit”) AND (“phone use” OR “screen time”)) |  |
|  | #24 | ((“RealizD” AND “app”) AND (“reduce” OR “limit”) AND (“phone use” OR “screen time”)) |  |
|  | #25 | ((“Stayfree” AND “app”) AND (“reduce” OR “limit”) AND (“phone use” OR “screen time”)) |  |
|  | #26 | ((“Off the Grid” AND “app”) AND (“reduce” OR “limit”) AND (“phone use” OR “screen time”)) |  |
|  | #27 | ((“Focus Me” AND “app”) AND (“reduce” OR “limit”) AND (“phone use” OR “screen time”)) |  |
|  | #28 | ((“Menthal” AND “app”) AND (“reduce” OR “limit”) AND (“phone use” OR “screen time”)) |  |
|  | #29 | ((“SocialX” AND “app”) AND (“reduce” OR “limit”) AND (“phone use” OR “screen time”)) |  |
|  | #30 | ((“My Phone Time” AND “app”) AND (“reduce” OR “limit”) AND (“phone use” OR “screen time”)) |  |
|  | #31 | ((“Minimalist Phone” AND “app”) AND (“reduce” OR “limit”) AND (“phone use” OR “screen time”)) |  |
|  | #32 | ((“FOMO” AND “app”) AND (“reduce” OR “limit”) AND (“phone use” OR “screen time”)) |  |
|  | #33 | ((“Lock Me Out” AND “app”) AND (“reduce” OR “limit”) AND (“phone use” OR “screen time”)) |  |
|  | #34 | ((“Focus Plant” AND “app”) AND (“reduce” OR “limit”) AND (“phone use” OR “screen time”)) |  |
|  | #35 | ((“Iron Will” AND “app”) AND (“reduce” OR “limit”) AND (“phone use” OR “screen time”)) |  |
|  | #36 | ((“Boring Phone” AND “app”) AND (“reduce” OR “limit”) AND (“phone use” OR “screen time”)) |  |
|  | #37 | ((“Focus Quest” AND “app”) AND (“reduce” OR “limit”) AND (“phone use” OR “screen time”)) |  |
|  | #38 | ((“Apprison” AND “app”) AND (“reduce” OR “limit”) AND (“phone use” OR “screen time”)) |  |
|  | #39 | ((“Dopamine Detox” AND “app”) AND (“reduce” OR “limit”) AND (“phone use” OR “screen time”)) |  |
|  | #40 | ((“DTox” AND “app”) AND (“reduce” OR “limit”) AND (“phone use” OR “screen time”)) |  |
|  | #41 | ((“Stay Blocked” AND “app”) AND (“reduce” OR “limit”) AND (“phone use” OR “screen time”)) |  |
|  | #42 | ((“YourHour” AND “app”) AND (“reduce” OR “limit”) AND (“phone use” OR “screen time”)) |  |
|  | #43 | ((“HelpMeFocus” AND “app”) AND (“reduce” OR “limit”) AND (“phone use” OR “screen time”)) |  |
|  | #44 | ((“Sma-Phospital” AND “app”) AND (“reduce” OR “limit”) AND (“phone use” OR “screen time”)) |  |
|  | #45 | ((“ScreenZen” AND “app”) AND (“reduce” OR “limit”) AND (“phone use” OR “screen time”)) |  |
|  | #46 | ((“OPAL” AND “app”) AND (“reduce” OR “limit”) AND (“phone use” OR “screen time”)) |  |
|  | #47 | ((“Screen Time” AND “app”) AND (“reduce” OR “limit”) AND (“phone use” OR “screen time”)) |  |
|  | #48 | ((“ActionFree” AND “app”) AND (“reduce” OR “limit”) AND (“phone use” OR “screen time”)) |  |
|  | #49 | ((“Offscreen” AND “app”) AND (“reduce” OR “limit”) AND (“phone use” OR “screen time”)) |  |
|  | #50 | ((“Attention” AND “app”) AND (“reduce” OR “limit”) AND (“phone use” OR “screen time”)) |  |
|  | #51 | ((“Digital distancing” AND “app”) AND (“reduce” OR “limit”) AND (“phone use” OR “screen time”)) |  |
|  | #52 | ((“Phonies” AND “app”) AND (“reduce” OR “limit”) AND (“phone use” OR “screen time”)) |  |
|  | #53 | ((“Overcome Phone Addiction” AND “app”) AND (“reduce” OR “limit”) AND (“phone use” OR “screen time”)) |  |
|  | #54 | ((“Timelimit.io” AND “app”) AND (“reduce” OR “limit”) AND (“phone use” OR “screen time”)) |  |
|  | #55 | ((“AddiLock” AND “app”) AND (“reduce” OR “limit”) AND (“phone use” OR “screen time”)) |  |
| GOOGLE SCHOLAR | #1 | ((“QualityTime” AND “app”) AND (“reduce” OR “limit”) AND (“phone use” OR “screen time”)) | 459 |
|  | #2 | ((“Detox Proc. Blocker” AND “app”) AND (“reduce” OR “limit”) AND (“phone use” OR “screen time”)) |  |
|  | #3 | ((“OFFTIME” AND “app”) AND (“reduce” OR “limit”) AND (“phone use” OR “screen time”)) |  |
|  | #4 | ((“SPACE” AND “app”) AND (“reduce” OR “limit”) AND (“phone use” OR “screen time”)) |  |
|  | #5 | ((“Forest” AND “app”) AND (“reduce” OR “limit”) AND (“phone use” OR “screen time”)) |  |
|  | #6 | ((“AppDetox” AND “app”) AND (“reduce” OR “limit”) AND (“phone use” OR “screen time”)) |  |
|  | #7 | ((“ActionDash” AND “app”) AND (“reduce” OR “limit”) AND (“phone use” OR “screen time”)) |  |
|  | #8 | ((“RescueTime” AND “app”) AND (“reduce” OR “limit”) AND (“phone use” OR “screen time”)) |  |
|  | #9 | ((“Digitox” AND “app”) AND (“reduce” OR “limit”) AND (“phone use” OR “screen time”)) |  |
|  | #10 | ((“Digital Wellbeing” AND “app”) AND (“reduce” OR “limit”) AND (“phone use” OR “screen time”)) |  |
|  | #11 | ((“iOS Screen Time” AND “app”) AND (“reduce” OR “limit”) AND (“phone use” OR “screen time”)) |  |
|  | #12 | ((“Flipd” AND “app”) AND (“reduce” OR “limit”) AND (“phone use” OR “screen time”)) |  |
|  | #13 | ((“AntiSocial” AND “app”) AND (“reduce” OR “limit”) AND (“phone use” OR “screen time”)) |  |
|  | #14 | ((“Social Fever” AND “app”) AND (“reduce” OR “limit”) AND (“phone use” OR “screen time”)) |  |
|  | #15 | ((“App Usage” AND “app”) AND (“reduce” OR “limit”) AND (“phone use” OR “screen time”)) |  |
|  | #16 | (My Addictometer” AND “app”) AND (“reduce” OR “limit”) AND (“phone use” OR “screen time”)) |  |
|  | #17 | ((“AppBlock” AND “app”) AND (“reduce” OR “limit”) AND (“phone use” OR “screen time”)) |  |
|  | #18 | ((“Stay Focused” AND “app”) AND (“reduce” OR “limit”) AND (“phone use” OR “screen time”)) |  |
|  | #19 | ((“Ubhind” AND “app”) AND (“reduce” OR “limit”) AND (“phone use” OR “screen time”)) |  |
|  | #20 | ((“Keep Me Out” AND “app”) AND (“reduce” OR “limit”) AND (“phone use” OR “screen time”)) |  |
|  | #21 | ((“Freedom” AND “app”) AND (“reduce” OR “limit”) AND (“phone use” OR “screen time”)) |  |
|  | #22 | ((“Daywise” AND “app”) AND (“reduce” OR “limit”) AND (“phone use” OR “screen time”)) |  |
|  | #23 | ((“Actuflow” AND “app”) AND (“reduce” OR “limit”) AND (“phone use” OR “screen time”)) |  |
|  | #24 | ((“RealizD” AND “app”) AND (“reduce” OR “limit”) AND (“phone use” OR “screen time”)) |  |
|  | #25 | ((“Stayfree” AND “app”) AND (“reduce” OR “limit”) AND (“phone use” OR “screen time”)) |  |
|  | #26 | ((“Off the Grid” AND “app”) AND (“reduce” OR “limit”) AND (“phone use” OR “screen time”)) |  |
|  | #27 | ((“Focus Me” AND “app”) AND (“reduce” OR “limit”) AND (“phone use” OR “screen time”)) |  |
|  | #28 | ((“Menthal” AND “app”) AND (“reduce” OR “limit”) AND (“phone use” OR “screen time”)) |  |
|  | #29 | ((“SocialX” AND “app”) AND (“reduce” OR “limit”) AND (“phone use” OR “screen time”)) |  |
|  | #30 | ((“My Phone Time” AND “app”) AND (“reduce” OR “limit”) AND (“phone use” OR “screen time”)) |  |
|  | #31 | ((“Minimalist Phone” AND “app”) AND (“reduce” OR “limit”) AND (“phone use” OR “screen time”)) |  |
|  | #32 | ((“FOMO” AND “app”) AND (“reduce” OR “limit”) AND (“phone use” OR “screen time”)) |  |
|  | #33 | ((“Lock Me Out” AND “app”) AND (“reduce” OR “limit”) AND (“phone use” OR “screen time”)) |  |
|  | #34 | ((“Focus Plant” AND “app”) AND (“reduce” OR “limit”) AND (“phone use” OR “screen time”)) |  |
|  | #35 | ((“Iron Will” AND “app”) AND (“reduce” OR “limit”) AND (“phone use” OR “screen time”)) |  |
|  | #36 | ((“Boring Phone” AND “app”) AND (“reduce” OR “limit”) AND (“phone use” OR “screen time”)) |  |
|  | #37 | ((“Focus Quest” AND “app”) AND (“reduce” OR “limit”) AND (“phone use” OR “screen time”)) |  |
|  | #38 | ((“Apprison” AND “app”) AND (“reduce” OR “limit”) AND (“phone use” OR “screen time”)) |  |
|  | #39 | ((“Dopamine Detox” AND “app”) AND (“reduce” OR “limit”) AND (“phone use” OR “screen time”)) |  |
|  | #40 | ((“DTox” AND “app”) AND (“reduce” OR “limit”) AND (“phone use” OR “screen time”)) |  |
|  | #41 | ((“Stay Blocked” AND “app”) AND (“reduce” OR “limit”) AND (“phone use” OR “screen time”)) |  |
|  | #42 | ((“YourHour” AND “app”) AND (“reduce” OR “limit”) AND (“phone use” OR “screen time”)) |  |
|  | #43 | ((“HelpMeFocus” AND “app”) AND (“reduce” OR “limit”) AND (“phone use” OR “screen time”)) |  |
|  | #44 | ((“Sma-Phospital” AND “app”) AND (“reduce” OR “limit”) AND (“phone use” OR “screen time”)) |  |
|  | #45 | ((“ScreenZen” AND “app”) AND (“reduce” OR “limit”) AND (“phone use” OR “screen time”)) |  |
|  | #46 | ((“OPAL” AND “app”) AND (“reduce” OR “limit”) AND (“phone use” OR “screen time”)) |  |
|  | #47 | ((“Screen Time” AND “app”) AND (“reduce” OR “limit”) AND (“phone use” OR “screen time”)) |  |
|  | #48 | ((“ActionFree” AND “app”) AND (“reduce” OR “limit”) AND (“phone use” OR “screen time”)) |  |
|  | #49 | ((“Offscreen” AND “app”) AND (“reduce” OR “limit”) AND (“phone use” OR “screen time”)) |  |
|  | #50 | ((“Attention” AND “app”) AND (“reduce” OR “limit”) AND (“phone use” OR “screen time”)) |  |
|  | #51 | ((“Digital distancing” AND “app”) AND (“reduce” OR “limit”) AND (“phone use” OR “screen time”)) |  |
|  | #52 | ((“Phonies” AND “app”) AND (“reduce” OR “limit”) AND (“phone use” OR “screen time”)) |  |
|  | #53 | ((“Overcome Phone Addiction” AND “app”) AND (“reduce” OR “limit”) AND (“phone use” OR “screen time”)) |  |
|  | #54 | ((“Timelimit.io” AND “app”) AND (“reduce” OR “limit”) AND (“phone use” OR “screen time”)) |  |
|  | #55 | ((“AddiLock” AND “app”) AND (“reduce” OR “limit”) AND (“phone use” OR “screen time”)) |  |
