# Supplementary material for: Evaluating the Effectiveness of Apps Designed to Reduce Mobile Phone Use and Prevent Maladaptive Mobile Phone Use: Multimethod Study
Source: J Med Internet Res. 2023 Aug 29;25:e42541. doi: 10.2196/42541 (PMC10498313; doi:10.2196/42541)
Supplement: Multimedia Appendix 3 [file jmir_v25i1e42541_app3.docx]

| **Section and Topic** | **Item #** | **Checklist item** | **Location where item is reported** |
| --- | --- | --- | --- |
| **INTRODUCTION** | | |  |
| Rationale | 1 | Describe the rationale for the review in the context of existing knowledge. | Page 2 |
| Objectives | 2 | Provide an explicit statement of the objective(s) or question(s) the review addresses. | Page 2 |
| **METHODS** | | |  |
| Eligibility criteria | 3 | Specify the inclusion and exclusion criteria for the review. | Page 3 |
| Information sources | 4 | Specify all databases searched to identify studies. | Figure 2 and Multimedia Appendix 2 |
| Search strategy | 5 | Present the full search strategies for all databases, including any filters and limits used. | Multimedia Appendix 2 |
| Selection process | 6 | Specify the methods used to decide whether a study met the inclusion criteria of the review, including how many reviewers screened each record and each report retrieved. | Page 3-4 |
| Data collection process | 7 | Specify the methods used to collect data from papers. |  |
| Data items | 8 | List and define all outcomes for which data were sought. | Multimedia Appendix 4 |
| Study risk of bias assessment | 9 | Specify the methods used to assess risk of bias in the included studies, including details of the tool(s) used, and how many reviewers assessed each study. | Multimedia Appendix 4 |
| Synthesis methods | 10 | Describe the processes used to decide which studies were eligible for each synthesis. | Multimedia Appendix 4 |
| **RESULTS** | | |  |
| Study selection | 11 | Describe the results of the search and selection process, from the number of records identified in the search to the number of studies included in the review, ideally using a flow diagram. | Figure 2 |
| Study characteristics | 12 | Cite each included study and present its characteristics. | Multimedia Appendix 4 |
| **DISCUSSION** | | |  |
| Discussion | 13a | Provide a general interpretation of the results in the context of other evidence. | Page 9-10 |
|  | 13b | Discuss any limitations of the evidence included in the review. |  |
|  | 13c | Discuss implications of the results for practice, policy, and future research. |  |

*From:*  Page MJ, McKenzie JE, Bossuyt PM, Boutron I, Hoffmann TC, Mulrow CD, et al. The PRISMA 2020 statement: an updated guideline for reporting systematic reviews. BMJ 2021;372:n71. doi: 10.1136/bmj.n71

For more information, visit: <http://www.prisma-statement.org/>
