# Supplementary material for: Evaluating the Effectiveness of Apps Designed to Reduce Mobile Phone Use and Prevent Maladaptive Mobile Phone Use: Multimethod Study
Source: J Med Internet Res. 2023 Aug 29;25:e42541. doi: 10.2196/42541 (PMC10498313; doi:10.2196/42541)
Supplement: Multimedia Appendix 4 [file jmir_v25i1e42541_app4.docx]

| **Country**  **Multimedia Appendix 4.** Summary of published literature evaluating the effectiveness of apps | **Included reviewed apps** | **Methods** | **Participant characteristics** | **Intervention** | **Findings** | **Effectiveness** | | **Global rating (EPHPP^a^)** | **Study** |  |
| --- | --- | --- | --- | --- | --- | --- | --- | --- | --- | --- |
|  |  |  |  |  |  | Mobile phone use | MMPU |  |  |  |
|  | | | | | | | | | | |
| United States | *Screen Time* (iOS) | Study design: cross-sectional study; participants were challenged to change their smartphone’s color setting to grayscale mode, turn off social media notifications, remove social media icons from the smartphone home screen, and place the device away from the bed when sleeping; MMPU scale: —^b^ | n=181, age: — (mean 25, SD —, years), sex: female: 67% and male: 33% | Three weeks grayscale mode | There is a reduction in mobile phone and social media use because of the grayscale mode (mean, SD, P —) | ↓^c^ | — ^b^ | Weak | Myers et al [11] |  |
| United Kingdom | *RescueTime* | Study 1 design: experimental study; *RescueTime* measures time spent on social networking sites (ie, app use, number of hours spent and productivity pulse); participants were granted access to *RescueTime* and received daily telephone calls asking them to estimate their daily mobile phone use; study 2 design: experimental study; *RescueTime* measures time spent on social networking sites (ie, app use, number of hours spent and productivity pulse); participants were granted access to *RescueTime* and received an SMS text message sent randomly during working hours; study 3 design: interview; participants were interviewed for 30 to 60 minutes to identify barriers to engagement and explore whether and how *RescueTime* leads to reflection and greater productivity; MMPU scale: — | Study 1: n=16, age: 22 to 28 (mean 23.75, SD 1.76) years, sex: female: 43.75% and male: 56.25%; study 2: n=30, age: 18 to 25 (mean 20.33, SD 1.54) years, sex: female: 60% and male: 40%; study 3: n=7, age: 25 to 40 (mean —, SD —) years, sex: female: 57.14% and male: 56.9% | Study 1: all participants used the app for 2 weeks baseline and 2 weeks follow-up; study 2: all participants used the app for 2 weeks of the experiment, but the control group did not receive questions via SMS text message; study 3: interview | Study 1: users were not engaged with the app, and *RescueTime* did not significantly reduce mobile phone use; study 2: users were not engaged with the app, and *RescueTime* did not significantly reduce mobile phone use (mean, SD, P — ); study 3: users were not engaged with *RescueTime* because of the lack of direction, information presented without explanation or context, and no advice | — | — | Weak | Collins et al [37] |  |
| Switzerland | *Screen Time* (iOS) | Study design: RCT^d^ and interview; participants were randomly assigned to the following conditions: grayscale mode, move an app, or the control group; the experiment lasted a week, during which participants came twice to the laboratory (ie, once at the beginning of the experiment and once at the end); use data were collected from the week preceding the experiment during the first laboratory meeting, and during the second meeting, use data from the intervention week were collected; during the intervention week, participants had to complete a daily questionnaire; a semi structured interview was conducted with the participants at the end of the study; MMPU scale: Mobile Phone Problematic Usage Scale developed by Bianchi and Phillips [50] | n=97, age: 18 to 29 (mean 21.41, SD 2.18) years, sex: female 81.4% and male: 18.6% | Duration: 1 week; move an app group (n=32), grayscale mode group (n=37), and control group (n=28) | Participants significantly reduced their objective mobile phone use compared with the week before the intervention (P<.05), but their self-reported MMPU increased (P<.05); there was no significant difference among the move an app group, grayscale mode group, or control group regarding reduction in mobile phone use (P>.05) (mean, SD —) | ↓ | ↑^e^ | Moderate | Ochs and Sauer [32] |  |
| Spain | *Screen Time* (iOS) and *QualityTime* | Study design 1 longitudinal field study; participants completed 2 surveys about mobile phone use before and after a workshop; in the intervening time, participants completed a mobile phone project in 6 to 25 days that comprised tracking mobile phone use, submitting the screenshots, and reporting 3 times per week; participants also evaluated the tracking experience and whether tracking had changed their mobile behavior; study design 2: web-based experiment survey; participants completed an experiment in the form of a survey about perceptions of app features; participants evaluated 3 apps in a within-participants design (informational tracking app vs grayscale mode digital nudge app vs coercive blocking app; counterbalanced); MMPU scale: — | *Study 1*: n: 280, age: — (mean 26; SD —) years, sex: female: 58.93% and male: 41.07%; *study 2*: n: 139, age: — (mean 28, SD 8.75) years, sex: female: 44.6% and male: 55.4% | *S*tudy 1: duration: 6 to 25 days of tracking mobile phone use; *study 2*: — | Study 1: tracking mobile phone use did not reduce screen time (P=.056); *study 2*: blocking app was evaluated as superior (P<.001) to the tracking app (P<.001) and digital nudge (P<.001) (mean, SD —) | ↔^f^ | — | Weak | Zimmermann [38] |  |
| United States | *Screen Time* (iOS) | Study design: RCT; first laboratory visit: participants were randomly assigned to a control or experimental group; the research assistant retrieved data from their iPhone *Screen Time* feature; second laboratory visit: participants returned to the laboratory 8 to 10 days later, and those in the experimental group had their mobile phones switched back to default; MMPU scale: — | n=161, age: 18 to 25 (mean 18.70, SD 1.15) years, sex: female: 73.9% and male: 27.1% | Control group: n=88 and grayscale mode group: n=73; the average duration of time spent in the study was 8.36 (SD 0.7; range 8-10) days | Greyscale reduced mobile phone use up to 37.90 minutes each day (Pre: mean 255.34, SD 100.46; Post: mean 217.44, SD 94.90, P≤.001), social media (Pre: mean 103.58, SD 56.05; Post: mean 79.61, SD 52.93, P<.001) and internet browsing (Pre: mean 11.34, SD 9.17; Post: mean 7.47, SD 7.84, P<.01), but did not influence video screen time (Pre: mean 21.07, SD 34.97; Post: mean 15.02, SD 35.16, P>.05) | ↓ | — | Moderate | Holte and Ferraro [33] |  |
| United States | *Screen Time* (iOS) | Study design: Experimental study and cross-sectional survey; all participants came to the laboratory on 2 separate visits. After the researchers received baseline data, 63 participants had their mobile phone’s screen display changed to grayscale mode, and they were told not to switch it back until after their second visit 8 days later; at the second visit, the procedure was repeated; screen time included entertainment, social media, and video games; MMPU scale: Smartphone Addiction Scale Short Version developed by [52] | n=133, age: 18 to 23 (mean 19, SD 0.95) years, sex: female: 87.2% and male: 12.8% | Control group: n=70 and grayscale group: n=63; baseline: day 0, first laboratory visit: day 9 (8 days later), and second laboratory visit: day 17 (8 days later) | Participants who had their mobile phones in grayscale mode exhibited a significant decrease in mobile phone use (Pre: mean 254.6, SD 100.9; Post: mean 232.8, SD 95.8, P<.05) and MMPU during their second visit (Pre: mean 27.6, SD 6.12; Post: mean 25, SD 7.1, P<.001) | ↓ | ↓ | Moderate | Holte et al [39] |  |
| United States | *Screen Time* (iOS) | Study design: RCT; short term: survey 1: demographic questions; survey 2: participants were required to submit screenshots of their mobile phone use from the previous 7 days and answer questions about their ideal and predicted mobile phone use for the following week; in the intervention group, participants were instructed to apply “app limits” and set time limits for their apps; survey 3: the same procedure was followed; long term: survey 4: the same procedure was applied to measure the longer-term effect; MMPU scale: — | n=629, age: 18 to 48 (mean 23.4, SD 4.4) years, sex: female: 57% and male: 43% | Survey 1: n=629; survey 2: n_control_=259 and n_limit nudge_=261 (app limit feature); survey 3: n_control_=236 and n_limit nudge_=237; and survey 4: n_control_=232 and n_limit nudge_=231 | Surveys 1, 2, and 3: app limit feature can significantly decrease mobile phone use by 6.2% per day (P<.05) and Facebook use by 33.2% (P<.01); survey 4: app limit feature can significantly decrease Facebook use by 36.8% (P<.001) and Instagram use by 33.9% (P<.01) (mean, SD —) | Surveys 1, 2, and 3: ↓; survey 4: ↓ | Surveys 1, 2, and 3: —; survey 4: — | Surveys 1, 2, and 3: Strong; survey 4: Strong | Hoong [34] |  |
| Germany and Poland | *Screen Time* (iOS) | Study design: RCT; 1 group was given a 20-day theory-based intervention on goal-directed mobile phone use that was divided into five 4-day modules: observe (physical reactions, eg, posture and impulses of mobile phone checking behavior), reflect (understanding habit and MMPU), vision (exercise on mindfulness, committed action, and goal setting), plan (action and coping planning), and support (tips to support sustainable behavior); the control group was applying time-out for a minimum of 1 hour per day; participants filled a questionnaire at baseline, after the intervention (on the 21st day), and at follow-up (on the 42nd day); MMPU scale: Mobile Phone Problem Use Scale developed by [51] | n=232, age: ≥18 years, sex: — | Intervention duration was 20 days (five 4-day training modules on goal-directed mobile phone use; n=55), with 3-week follow-up (n=44); control group (time-out applied for at least 1 hour per day for 20 days; n=55), with 3-week follow-up (n=44) | Both theory-based intervention on goal-directed mobile phone use and time-out control strategy did not effectively reduce mobile phone use and MMPU (P=.55) | ↔ | ↔ | Moderate | Keller et al [35] |  |
| Canada | *Screen Time* (iOS) | Study design: experimental study; study 1: pre-post design; participants were asked to follow a 10-part behavioral intervention to reduce mobile phone use and MMPU; at the first laboratory visit, the *Screen Time* feature’s screenshot was submitted (baseline), followed by 2 weeks of the interventions; at the second laboratory visit, participants submitted a fresh screenshot and filled out the questionnaires; MMPU scale: Smartphone Addiction Scale Short Version [53]; study 2: Experimental study and interview; participants completed 2 laboratory visits over 2 weeks (same as study 1); control group: screen time and tracking alone and intervention group: full intervention (10 steps); the follow-ups were in the second week, fourth week, and sixth week; MMPU scale: Smartphone Addiction Scale Short Version developed by [52] | Study 1: n=51, age: 18 to 34 (mean 21.7, SD 2.8) years, sex: female: 71% and male: 29%; study 2: n=70, age: 18 to 33 (mean 20.7, SD 2.6) years, sex: female: 77% and male: 23% | There were 10 nudge intervention strategies: disable notifications, keep mobile phone on silent and out of reach, disable easy unlocking and use a password instead, enable sleep mode, enable grayscale mode, hide social media apps, use computers instead, let others know that you are taking a break from your mobile phone, leave the mobile phone at home, and minimize the use of the mobile phone; study 1: 2 weeks and study 2: 6 weeks | Study 1: the intervention can decrease mobile phone use (Pre: mean 4.67, SD 2.09; Post: mean 3.40, SD 1.99, P<.001) and MMPU(Pre: mean 35.29, SD 8.84; Post: mean 28.08, SD 9, P<.001); study 2: across conditions can decrease mobile phone use (Pre: mean 4.14, SD 1.59; Post: mean 3.19, SD 1.48, P<.01) and MMPU(Pre: mean 30.80, SD 7.84; Post: mean 25.31, SD 7.18, P<.001) | Study 1: ↓; study 2: ↓ | Study 1: ↓; study 2: ↓ | Moderate | Olson et al [40] |  |
| United States | *Screen Time* (iOS) | Study design: cross-sectional study; the survey included questions about the participants’ mobile phone use and the emotions they felt when using their mobile phones with time limit features MMPU scale: — | n=37, age: —, sex: — | — | No results were found in terms of effectiveness to reduce mobile phone use and MMPU (mean, SD, P—) | — | — | Weak | Prasad and Quinones [45] |  |
| China | *Screen Time*^g^ and *Forest* | Study design: semi structured in-depth web-based interviews; participants were interviewed about efforts to cut or control mobile phone use using apps; MMPU scale: — | n=70, age: —, sex: female: 74.29% and male: 25.71% | Participants were interviewed for 30 to 60 minutes | Of the 26 participants, 19 (73%) agreed that apps could reduce mobile phone use; however, the sample size of this study was minimal, and a firm conclusion cannot be drawn | ↓ | — | N/A^h^ (qualitative study) | Dai et al [43] |  |
| United Kingdom | *AntiSocial* | Study design: RCT; participants were instructed to use the *AntiSocial* app to self-monitor their mobile phone use and social media use and were also asked to block the app once the designated time limit was reached; concurrently, participants engaged actively with the Headspace app for 10 days, participating in brief mindfulness sessions, and used the Pacifica app for mood tracking; subsequently, participants were requested to complete online pre- and post-intervention assessments, specifically focusing on FOMO^i^ and Nomophobia; MMPU scale: Fear of Missing Out developed by [53] and NMP-Q^k^ developed by [54] | n 252, age: 18 to 32 (mean 20.72, SD 3.12) years, sex: female: 82% and male: 18% | Intervention duration was 10 days (n=123): self-monitoring mobile phone use (including mindfulness, mood tracking, receiving daily reminder email, and daily online blogging); control group (n=129) | There was a significant decrease in daily mobile phone use (Pre: mean 4.515, SD 2.28; Post: mean 3.51, SD 1.88, P<.001) and FoMO (Pre: mean 3.48, SD 1.36; Post: mean 2.86, SD 1.16, P<.001), but no significant results were found in Nomophobia (Pre: mean 77.17, SD 2.40; Post: mean 78.03, SD 2.72) | ↓ | FOMO: ↓; Nomophobia: ↔ | Moderate | Throuvala et al [36] |  |
| South Africa | *Screen Time* (iOS)*, Digital Wellbeing, Forest, AppBlock, Flipd,* and *AntiSocial* | Study design: cross-sectional study and interview; participants were divided into app users and nonapp users, and app users were asked to estimate mobile phone use during an average day, classify their engagement with the apps and features commonly used, describe their motivations to use the apps, and assign a perceived value (value of data provided, effectiveness regarding behavior change, and effectiveness regarding supporting productivity), whereas nonapp users were asked about their future use intentions; for the interviews, app users were invited and asked to describe their understanding of apps, the factors that have motivated or averted use, and the perceived outcomes of using apps; MMPU scale: — | n 1849, age: — (mean 20.53, SD 1.87) years, sex: female: 62.7% and male: 36.9%, interview: n: 332 | Intervention: the use of apps and features | Apps could help reduce excessive mobile phone use (not clear, mean 4.61, SD 1.65 on 7-point Likert scales, P —); the features used were weekly reports on overall use (65.7%) and use of specific apps (43.6%), disabling notifications (43.6%), daily overall use (41%) and daily use of specific apps (25%), timers restricting access to specific apps (36%) and restricting device access (26.4%), and blocking specific apps (18.3%) | — | — | Weak | Parry et al [44] |  |
| United States | *Screen Time* (iOS) | Study design: Cross-sectional study; participants who use the *Screen Time* (iOS) features (n=151) were asked to report mobile phone use recorded over the previous week and to answer questions related to the adoption of technology; MMPU scale: — | n=405, age: 19 to 81 (mean 38.27, SD 12.86) years, sex: female: 60.5% and male: 39.5% | — | No results found in terms of reducing mobile phone use and MMPU | — | — | Weak | Oeldorf-Hirsch and Chen [46] |  |
| Estonia | *App Usage* | Study design: experimental study; this study aimed to investigate the impact of tracking apps and pop-up notifications on reducing MMPU; all groups completed a problematic smartphone use measure (time 1), and, subsequently, the active and passive experimental groups used a smartphone tracking app for 3 weeks; after the data collection period, participants in the active experimental group received instructions on configuring their app to receive notifications based on their smartphone use patterns for the following month (time 2); by contrast, the passive experimental group continued tracking their smartphone use without receiving notifications; finally, all participants from the different groups were re-evaluated regarding their problematic smartphone use (time 3); MMPU scale: Smartphone Addiction Scale developed by [52] | n=73, age: 18 to 58 (mean 26.5, SD 8.5) years, sex: female: 82% and male: 18% | Three weeks: control, active experimental, and passive experimental; time 1, time 2 (3 weeks later), and time 3 (1 month later) | Tracking and pop-up notifications cannot reduce mobile phone use and MMPU(P=.309) | ↔ | ↔ | Weak | Loid et al [41] |  |
| Sweden | *AppDetox* | Study design: Observational research-secondary data; AppDetox offers 3 distinct rules (Forever, TimeOfDay, and Countdown) that assist users in limiting app use; in a real-world deployment of the app, researchers examined the types of rules users established to regulate their mobile phone use and how users occasionally violated their own rules; MMPU scale: — | n=11,700 (data collected from the app users); age: 18 to 58 (mean 26.5, SD 8.5) years, sex: female: 52% and male: 48% | — | Users set the rules for the day and mainly restrict the use of messaging apps  (mean, SD, P —) | — | — | Weak | Löchtefeld et al [49] |  |
| Germany | *Screen Time* (iOS)*,* *Forest*, *Digital Wellbeing*, *Detox ProcrastinationBlocker,* *Space*, and *Offtime* | Study design: Cross-sectional study; participants were asked to indicate their daily mobile phone use on social networking sites (ie, Facebook, Instagram, YouTube, Snapchat, and WhatsApp) and answer 4 questions related to MMPU; participants were divided into 2 groups: “digital detox” app users and nonusers; multigroup analysis was applied to determine the relationship between “digital detox” app users and nonusers regarding social media use and MMPU; MMPU scale: an adapted set of 4 questions used to measure MMPU based on the study by Roberts et al [55] | n: 500, age: 18 to 35 (mean 22.37, SD 3.47) years, sex: female: 57.8% and male: 42.2% | — | No relationship found between social networking sites and MMPU among “digital detox” app users (mean, SD, P —) | — | — | Weak | Schmuck [47] |  |
| United Kingdom | *RescueTime* | Study design: retrospective cross-sectional estimation study; the intervention lasted for 4 weeks, but the recording of mobile phone use began at the start of week 3; an interview was conducted at the beginning of week 3, followed by an exit interview at the end of week 4; these interviews aimed to explore participants’ attitudes toward social networks, assess their time management skills, and measure their perceived stress levels associated with social network use; MMPU scale: — | n: 16, age: 19 to 22 (mean 22.37, SD 3.47) years, sex: female: 57.8% and male: 42.2% | Two weeks: no records and 2 weeks: intervention (*RescueTime* app and Interview) | There was no significant effect on reducing time spent on social networking sites (mean —, SD —, P=.271) | ↔ | — | Weak | Zhou et al [48] |  |
| United States | *Flipd* | Study design: experimental study; participants received an individual user license for *Flipd* app, which was linked to the specific course they were enrolled in, and were motivated to self-monitor and actively reduce mobile phone use and distractions during the 90-minute class sessions throughout the 16-week semester; in addition, they had access to weekly mobile phone distraction statistics, the ability to set personal daily reminders, and feedback on their classroom mobile phone use; these features served as additional proactive and reactive notification prompts | n: 234, age: —, sex: — | Control group: 3 classes and intervention group: 2 classes with 4 phases: phase 1: baseline (none using *Flipd* app), phase 2: intervention to monitor the effects of the “reminder prompt” (participant’s natural tendency to use *Flipd* app), phase 3: another “reminder prompt” after midterms examinations was provided as a probe, and phase 4: another assessment to monitor the effects of the “reminder prompt” until the end of the semester | No result can be found in terms of reduction on mobile phone use and MMPU; however, this research showed (1) a positive linear relationship between *Flipd* app use and attendance rates (*r*=0.360; *P*<.001; *r*^2^=0.600 [effect size]), (2) a negative linear relationship between *Flipd* app use and ≥3 absence rates (*r*=−0.340; *P*<.001; *r*^2^=0.587 [effect size]), (3) 10% of the students resisted using it, and (4) a reduction of 14.34% of mobile phone distractions within the classroom | — | — | Weak | Neuwirth [42] |  |

^a^EPHPP: Effective Public Health Practice Project.

^b^—: Not available.

^c^↓: decrease.

^d^RCT: randomized controlled trial.

^e^↑: increase.

^f^↔: no change.

^g^*Screen Time* (app) is different from *Screen Time* (iOS).

^g^N/A: not applicable.

^h^FoMO: Fear of Missing Out.

^i^NMP-Q: Nomophobia Questionnaire.
