# Supplementary material for: Evaluating the Effectiveness of Apps Designed to Reduce Mobile Phone Use and Prevent Maladaptive Mobile Phone Use: Multimethod Study
Source: J Med Internet Res. 2023 Aug 29;25:e42541. doi: 10.2196/42541 (PMC10498313; doi:10.2196/42541)
Supplement: Multimedia Appendix 5 [file jmir_v25i1e42541_app5.docx]

**Multimedia Appendix 5.** Search terms in a custom topic for user reviews.

| **Custom topic** | **Search terms** |
| --- | --- |
| Reduce phone use and MMPU level | Reduce OR effective OR control OR accurate OR track OR block OR lock OR goal OR focus OR useful OR rules OR limit OR help OR set OR concentrate OR work OR ineffective OR break OR habit |
| Tracking | Track OR history OR notif* OR notification OR visual* OR alert OR warn* OR progress OR comparison OR usage OR report OR launch OR unlock |
| Easy to use | Easy OR use OR useful |
| Blocking | Block OR lock OR schedule offtime OR offtime OR focus OR strict mode OR do not disturb OR stop disturb OR reduce distract* OR password OR PIN OR auto-lock |
| Goal setting | Goal OR set time OR set goal OR motivate* OR motivational quotes OR set rule OR focus |
| Notification | Notif* OR alert OR warn* OR remind* |
| Gamification | Real world OR tree OR grow OR plant OR reward |
| Multiple devices | PC OR tablet OR other devices OR multiple devices |
| Global comparison | Global OR comparison OR addiction level OR assessment |
| Grayscale | Gray OR grey OR grayscale OR greyscale OR black white OR wind down OR bedtime |
| Motivational quotes | Motivate* OR motivation OR motivational OR write reason OR |
